# Bayesian inference for identifying tumour-specific cancer dependencies through integration of ex-vivo drug response assays and drug-protein profiling

**DOI:** 10.1186/s12859-024-05682-0

**Published:** 2024-03-08

**Authors:** Hanwen Xing, Christopher Yau

**Affiliations:** 1 Nuffield Department of Women’s and Reproductive Health, University of Oxford, Oxford, UK; 2 Health Data Research UK, London, UK

**Keywords:** Tumor-specific molecular dependencies, Chemical perturbation, Gaussian process, Spike-and-slab regression

## Abstract

The identification of tumor-specific molecular dependencies is essential for the development of effective cancer therapies. Genetic and chemical perturbations are powerful tools for discovering these dependencies. Even though chemical perturbations can be applied to primary cancer samples at large scale, the interpretation of experiment outcomes is often complicated by the fact that one chemical compound can affect multiple proteins. To overcome this challenge, Batzilla et al. (PLoS Comput Biol 18(8): e1010438, 2022) proposed DepInfeR, a regularized multi-response regression model designed to identify and estimate specific molecular dependencies of individual cancers from their ex-vivo drug sensitivity profiles. Inspired by their work, we propose a Bayesian extension to DepInfeR. Our proposed approach offers several advantages over DepInfeR, including e.g. the ability to handle missing values in both protein-drug affinity and drug sensitivity profiles without the need for data pre-processing steps such as imputation. Moreover, our approach uses Gaussian Processes to capture more complex molecular dependency structures, and provides probabilistic statements about whether a protein in the protein-drug affinity profiles is informative to the drug sensitivity profiles. Simulation studies demonstrate that our proposed approach achieves better prediction accuracy, and is able to discover unreported dependency structures.

**Supplementary Information:**

The online version contains supplementary material available at 10.1186/s12859-024-05682-0.

## Introduction

The abnormal pathway activities due to genetic or epigenetic changes are often responsible for the continuous growth or apoptosis resistance in cancer cells. However, the specific molecular mechanisms driving these activities differ widely among various cancer types and individual patients. Such diversity in the molecular mechanisms can result in varying responses to treatment outcomes. The key objective of precision cancer therapy is to exploit this diversity and identify the inherent weaknesses unique to each tumor.

Novel precision cancer therapies rely on the identification of potentially druggable targets via e.g. cancer dependency mapping. Genetic perturbations such as RNAi and CRISPR/Cas9 systems offer robust and scalable strategies for pinpointing cancer-specific dependencies. These methods manipulate genes using techniques like RNA interference (RNAi) and the CRISPR/Cas9 system to suppress or eliminate particular genes within cancer cells, and reveal the subsequent impact on cell growth or survival. Although RNAi and CRISPR/Cas9 are widely applicable, their application becomes challenging when working with primary tumor samples in the context of clinical research. In addition, genetic manipulations and the targeted inhibition of the protein produced by the gene may not necessarily lead to the same effect. Such discrepancies could be attributed to the fact that drug molecules might quantitatively impede the enzymatic function of a protein while leaving other functions untouched, while genetic manipulation may affect all of its functions simultaneously.

Compared with genetic perturbation methods such as RNAi and CRISPR/Cas9 systems, chemical perturbation experiments, which involve high-throughput screening of bioactive compounds on cancer cells, offer an appealing alternative for personalized oncology. These experiments are well-suited for primary tumor models, enabling the recognition of patient- and tumor-specific dependencies. However, a significant challenge arises from the polypharmacological nature of small compounds, as many exhibit diverse off-target effects, hindering the identification of druggable protein targets associated with the desired outcome.

To better utilise high-throughput drug sensitivity datasets for precision medicine, Batzilla et al. [3] proposed DepInfer, a regularized multi-response regression model designed to identify and estimate unobserved, specific molecular dependencies of individual cancers from their ex-vivo drug sensitivity profiles obtained from chemical perturbation experiments. The authors demonstrated that DepInfer are able to correctly identify known kinase dependencies of individual cancers in multiple real-world datasets. However, DepInfer does not provide uncertainty estimates on either the in- or exclusion of the molecular dependencies, or the size of them, which are crucial for users’ decision-making process. In addition, DepInfeR imputes missing values in protein-drug affinity and drug sensitivity profiles by either filling in the missing values manually or using a single imputation step. Such imputation steps could lead to potentially biased and overly confident results [22]. These limitations affect the utility and feasibility of DepInfeR.

In this paper, we propose a Bayesian extensions of DepInfeR to address its limitations. Compared with DepInfeR, our proposed method is able to capture potentially non-linear dependency structures between the proteins and the samples using Gaussian process, and allow users to make probabilistic statements about whether or not such dependencies are supported by the dataset. In addition, our methods handle missing values in both protein-drug affinity and drug sensitivity profiles in an automatic fashion, which minimizes user inputs and improves the robustness of the method. To demonstrate the efficacy of our method, we applied the proposed method to the same datasets used in Batzilla et al. [3]. Simulation results show that our method consistently outperforms DepInfeR in term of prediction accuracy, and are able to identify multiple known kinases dependencies that were not picked up by DepInfeR. Furthermore, our method also detects previously unreported dependencies between kinases and cancer cells in the same datasets analyzed by Batzilla et al. [3]. These findings further highlight the utility of our proposed method in revealing insights of patient or cancer type specific pharmacological intervention.

## Background

We start by fixing the notations. Let *D*, *P*, *S* be the number of drugs, proteins, and cell samples respectively. Let $\varvec{X}$ be the $D \times P$ processed drug-protein affinity matrix with each entry $X_{dp}$ being the processed drug-protein affinity score of the *d*th drug on the *p*th protein for $d=1,\dots ,D; \ p=1,\dots ,P$. Similarly, let $\varvec{Y}$ be the $D \times S$ processed drug-sensitivity matrix with each entry $Y_{ds}$ being the processed sensitivity measure of the *d*th drug on the *s*th sample for $d=1,\dots ,D; \ s=1,\dots ,S$.

Batzilla et al. [3] proposed DepInfeR, a regularized multivariate linear regression model, to identify and estimate the protein-sample dependence: Let $\textbf{1}_D$ being a *D*-dimensional column vector of ones. The authors proposed

1 $$\begin{aligned} \varvec{Y} = \varvec{\beta _0} + \varvec{X} \varvec{\beta } + \varvec{\epsilon }, \end{aligned}$$

where $\varvec{\beta _0} = [\beta _{01}\textbf{1}_D,\ldots ,\beta _{0\,S}\textbf{1}_D]\in \mathbb {R}^{D\times S}$ is the intercept matrix with $\beta _{01},\ldots ,\beta _{0S} \in \mathbb {R}$, $\varvec{\beta } \in \mathbb {R}^{P\times S}$ is the regression coefficient matrix and $\varvec{\epsilon } \in \mathbb {R}^{D\times S}$ is the residual matrix. The estimated parameter matrices $\hat{\varvec{\beta }}_0$ and $\hat{\varvec{\beta }}$ in DepInfeR are obtained by repeatedly fitting a multi-response Gaussian linear model with group-LASSO regularization [2, 28] under different penalty parameters, recording the fitted parameter matrices, and finally taking the element-wise median of the fitted parameter matrices. Given the fitted parameter matrices, the model defined in Eq (1) implies that the sensitivity measure $Y_{ds}$ of the *d*th drug on the *s*th sample can be written as

2 $$\begin{aligned} Y_{ds} = \hat{\beta }_{0s} + \sum _{p=1}^P X_{dp} \hat{\beta }_{ps} + \hat{\epsilon }_{ds}, \end{aligned}$$

where $\hat{\beta }_{0s}$ is the fitted intercept, $\hat{\varvec{\epsilon }} = \varvec{Y}-\varvec{X}\hat{\varvec{\beta }}$ is the fitted residual matrix, and $Y_{ds}, X_{dp}, \hat{\beta }_{ps}, \hat{\epsilon }_{ds}$ are the corresponding entries in $\varvec{Y},\varvec{X},\hat{\varvec{\beta }},\hat{\varvec{\epsilon }}$ respectively. A non-zero entry $\hat{\beta }_{ps}$ in $\hat{\varvec{\beta }}$ encodes the direction and magnitude of the (additive) contribution of the *p*th protein to the *s*th sample, as the model assumes that for the *s*th sample, the contribution of the *p*th protein to the sensitivity measure $Y_{ds}$ is a linear function of the affinity score $X_{dp}$ with $\hat{\beta }_{ps}$ being the slope for all $d=1,\ldots D$. The sparsity of group-LASSO ensures that the estimated parameter matrix $\hat{\beta }$ would consist of rows of zeros, which can be viewed as proteins that do not contribute to the sensitivity measure at all (i.e. not selected by the model).

### Limitations of DepInfeR

Batzilla et al. [3] demonstrated that DepinfeR is able to correctly identify known protein-cell sample dependencies in multiple datasets. However, DepInfeR has a few limitations. First, DepInfeR handles missing values in $\varvec{Y}$ by filling the missing entries using random forest imputation [24]. This single imputation step does not account for the uncertainty in predicting the missing values, and could lead to bias in the regression analysis [20, 22]. Secondly, even though the sparsity of group LASSO in DepInfeR helps users to identify and select relevant proteins, it is not able to provide uncertainty estimates of the inclusion or exclusion of a protein, which is crucial for selecting the subset of relevant proteins. Thirdly, the estimated parameter matrix $\hat{\varvec{\beta }}$ in DepInfeR is obtained by taking element-wise median. This may improve the robustness of the estimator, but it also complicates the uncertainty estimation of the parameters, and affects the fitting of the model (See Additional file 1: Sect. 1.1). In the following section, we propose a Bayesian extensions of DepInfeR that address these limitations.

In addition, DepinfeR recommends normalizing the processed drug-sensitivity matrix $\varvec{Y}$ using *z*-scores. This data-dependent normalization step does not always respect the model assumption, and may affect prediction performance. In “GDSC1”, “BeatAML” and “EMBL” sections, we also demonstrate how data-independent transformations such as logit or log transformation can lead to better prediction accuracy.

## Spike-and-slab Gaussian process regression

To address the limitations of DepInfeR discussed in the last section, we propose a Bayesian extension of DepInfeR using a spike-and-slab Gaussian process regression model. DepInfeR assumes that for each sample *s* and the drug *d*, the contribution of each protein *p* to the sensitivity measure $Y_{ds}$ is a linear function of the corresponding drug-protein affinity score $X_{dp}$. This assumption may not be flexible enough to capture the reality. Hence in this paper, we extend DepInfeR using Gaussian Processes to model the protein-cell sample dependencies, allowing the model to adapt to more complex non-linear molecular dependency structures. We also considered a similar but less flexible linear version of the proposed model, which shares the same linear assumption as in DepInfeR (see Additional file 1: Sect. 2).

Let $a_0, b_0 > 0$, $\pi _0 \in (0,1)$. Let $k_\nu (\cdot , \cdot ): \mathbb {R} \times \mathbb {R} \rightarrow \mathbb {R}$ be a valid kernel function with hyper parameter $\nu$. We define the Spike-and-Slab Gaussian process model as follows:

3 $$\begin{aligned} z_p \sim \text {Bernoulli}(\pi _0), \ p=1,\dots ,P; \end{aligned}$$

4 $$\begin{aligned} \quad \sigma ^2 \sim \text{ Inv-Gamma }(a_0,b_0); \end{aligned}$$

5 $$\begin{aligned} f_{ps}: \mathbb {R} \rightarrow \mathbb {R} \sim \mathcal{G}\mathcal{P}(0, k_\nu ), \ p=1,\dots ,P; \ s=1,\dots ,S; \end{aligned}$$

6 $$\begin{aligned} \gamma ^2 \sim \text{ Half-Normal }(0, 1), \ a_s|\gamma ^2 \sim N(0, \gamma ^2), \ s=1,\dots ,S; \end{aligned}$$

7 $$\begin{aligned} \epsilon _{ds} \sim N(0, \sigma ^2), \ s=1,\dots ,S; \ d=1,\dots ,D; \end{aligned}$$

8 $$\begin{aligned} Y_{ds} = a_s + \sum _{p=1}^P z_p f_{ps}(X_{dp}) + \epsilon _{ds}, \ s=1,\dots ,S; \ d=1,\dots ,D. \end{aligned}$$

**Fig. 1:**
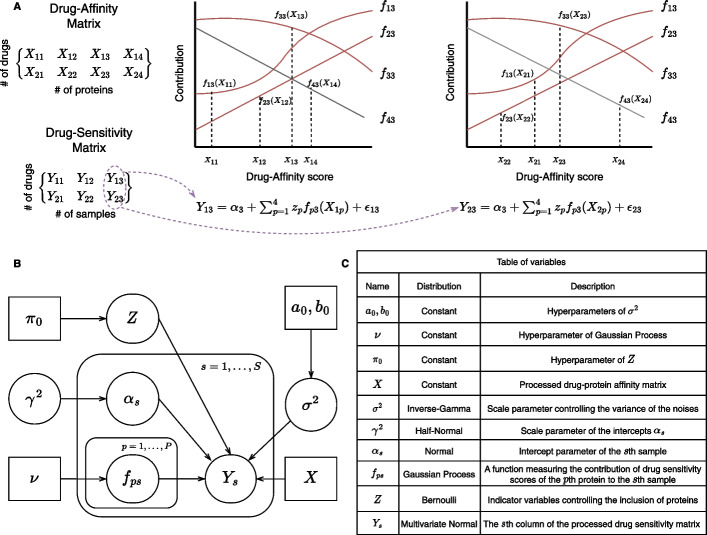
**A** A schematic illustration of how the $s=3$rd column of the drug-sensitivity matrix $\varvec{Y}$ is generated under the proposed Gaussian Process regression model using the set of functions $\{f_{ps}\}_{p=1}^4$. Here we set $Z=\{1,1,1,0\}$. This means the $p=4$th protein does not contribute to the sensitivity measure, and the corresponding function $f_{43}$ is colored in grey. **B** Graphical representation of the proposed Spike-and-Slab Gaussian Process regression model. **C** A table of all variables used to define the proposed model

The binary variables $z_p$ controls the inclusion of the *p*th protein (note that $z_p$ excludes proteins in a similar fashion to the group-LASSO penalty used in DepInfeR: when $z_p=0$, $Y_{ds}$ does not depend on the protein-drug affinity score $X_{dp}$ *for all* $d=1,\dots ,D$). The scalar parameter $\alpha _s$ is the intercept parameter of the *s*th column of $\varvec{Y}$, and $\sigma ^2$ controls the scale of the Gaussian noises $\epsilon _{ds}$. This proposed approach shares the same additive structure as DepInfeR, but we now model the contribution of the *p*th protein to the *s*th sample as a *random function* $f_{ps}$ of the drug affinity scores. In contrast, DepInfeR assumes that the contribution of the *p*th protein to the *s*th sample is linear with slope $\beta _{ps}$. Using Gaussian Process as a non-linear regression model greatly improves the flexibility of the model, and allows the model to identify more complex molecular dependency structures. See also Fig. 1 for a schematic illustration of the proposed model.

We now discuss the choice of the kernel function $k_\nu$. From Eqn (2) we see the linear assumption in DepInfeR implies that when $X_{dp}=0$, the *p*th protein does not contribute to the sensitivity measure $Y_{ds}$ for any $s=1,\dots ,S$ regardless of the value of $\hat{\beta }_{ps}$. This is a natural constraint: When $X_{dp}=0$, we expect this protein to have no contribution to the sensitivity measure as the drug would simply not bind to this protein. This observation implies that in our setup, the individual contribution functions should satisfy $f_{ps}(0) = 0$ for all *p*, *s*. Let $\mathcal{G}\mathcal{P}(0, k_{\nu })$ be a Gaussian process with zero mean function and an arbitrary kernel $k_{\nu }$. Instead of sampling $f_{ps}$ from the original $\mathcal{G}\mathcal{P}(0, k)$, we can impose this functional constraint by sampling $f_{ps}$ from a *conditional* Gaussian process $f_{ps}|f_{ps}(0)=0$: By standard properties of Gaussian process [16], it is straightforward to show that this conditional Gaussian process $f_{ps}|f_{ps}(0)=0$ built on $\mathcal{G}\mathcal{P}(0, k_{\nu })$ is itself a zero-mean Gaussian process, and its kernel function $k_\nu ^{(0)}$ takes the form

9 $$\begin{aligned} k_\nu ^{(0)}(x_1,x_2) = k_\nu (x_1,x_2) -k_\nu (x_1, 0) k_\nu (x_2, 0)k_\nu (0,0)^{-1}. \end{aligned}$$

As a result, any random function $f_{ps} \sim \mathcal{G}\mathcal{P}(0, k_\nu ^{(0)})$ would satisfy the constraint $f_{ps}(0)=0$. We suggest using this modified kernel $k_\nu ^{(0)}$ instead of the original $k_\nu$ whenever possible as it respects this particular aspect of the underlying physical process of the experiment. An example of $k_\nu ^{(0)}(x_1,x_2)$ will be given in “Posterior inference” section.

### Missing values in *X* and *Y*

DepInfeR handles missing values in $\varvec{Y}$ using Random-Forest-based single imputation, which does not account for the uncertainty in the prediction of the missing values. In contrast, our proposed model is able to handle missing values in $\varvec{Y}$ in a statistically more principled way: Under the Bayesian framework, we are able to view missing values in $\varvec{Y}$ as unobserved random variables (i.e. addition model parameters). Specifically, suppose $Y_s$, the *s*th column of $\varvec{Y}$, consists of multiple missing values. Let $M_s$ be the set of indices whose corresponding entries in $Y_s$ is missing. Let $Y_{M_s}$ and $\varvec{Y}_{-M_{s}}$ be the missing and observed entries of $Y_s$ respectively. It is straightforward to see that $\varvec{Y}_{M_s}$ and $\varvec{Y}_{-M_{s}}$ are conditionally independent given the model parameters for any $s=1,\dots ,S$. Therefore instead of imputing the missing values directly, we can easily incorporate the additional uncertainty introduced by missing values by first marginalizing the unobserved part out from the likelihood function conditioned on all model parameters, and then carrying out posterior inference conditioned solely on the observed values $\varvec{Y}_{obs} = \{\varvec{Y}_{-M_s}\}_{s=1}^S$ thanks to the conditional independent assumption.

Before we give the likelihood of the observed $Y_{obs}$, we also need to address the missing values in the drug-affinity matrix $\varvec{X}$. In practice, $\varvec{X}$ consists a large number of missing entries. For example, approximately $90\%$ of entries in the raw $\varvec{X}$ matrix of the GDSC1 dataset used in Batzilla et al. [3] are missing. In Batzilla et al. [3], the authors filled all missing values in the raw drug-affinity matrix manually without justification. In this paper, we consider a different assumption that, for the *d*th drug and *p*th protein, if the corresponding drug-affinity score $X_{pd}$ is missing, then the *p*th protein simply does not contribute to the sensitivity measure $Y_{ds}$ for all samples $s=1,\dots ,S$. If we impose this assumption on our proposed model, then each sensitivity measure $Y_{ds}$ in (8) would then follow

10 $$\begin{aligned}&Y_{ds} = \mu _{ds} + \epsilon _{ds}; \end{aligned}$$

11 $$\begin{aligned}&\mu _{ds}=a_s + \sum _{p=1}^P z_p \mathbbm {1}(X_{dp} \text{ not } \text{ missing})f_{ps}(X_{dp}) \end{aligned}$$

for $s=1,\ldots ,S$, $d=1,\ldots ,D$ where $\mathbbm {1}(\cdot )$ is the indicator function. Let $\varvec{\mu }^{(s)} = \{\mu _{ds}\}_{d=1}^D$, $\varvec{Z} = \{z_p\}_{p=1}^P$ and $\varvec{\alpha }=\{\alpha _s\}_{s=1}^S$. Under this assumption, the likelihoods of the observed column $\varvec{Y}_{-M_s}$ and the full observed dataset $\varvec{Y}_{obs} = \{\varvec{Y}_{-M_s}\}_{s=1}^S$ given the drug-affinity matrix $\varvec{X}$ and all model parameters are then

12 $$\begin{aligned} p\left( \varvec{Y}_{-M_s}|\varvec{X}, \alpha _s, \{f_{ps}\}_{p=1}^P, Z, \sigma ^2\right) = \mathcal {N}\left( \varvec{Y}_{-M_s}; \varvec{\mu }^{(s)}_{-M_s}, \sigma ^2 \varvec{I}_{D-|M_s|}\right) \end{aligned}$$

and

13 $$\begin{aligned} p\left( \varvec{Y}_{obs}|\varvec{X}, \varvec{\alpha }, \{f_{ps}\}_{s,p=1}^{S,P}, \varvec{Z}, \sigma ^2\right) = \prod _{s=1}^S p\left( \varvec{Y}_{-M_s}|X, \alpha _s, \{f_{ps}\}_{p=1}^P, \varvec{Z}, \sigma ^2\right) \end{aligned}$$

respectively, where $\varvec{I}_D$ is a $D\times D$ identity matrix and $\mathcal {N}(\cdot ; \mu , \Sigma )$ is the multivariate Gaussian density with mean $\mu$ and covariance matrix $\Sigma$. In the following section, we will carry out posterior inference of the model parameters using the likelihood given above. From simulation studies in “GDSC1”, “BeatAML” and “EMBL” sections we find that this pre-processing procedure, combined with the proposed model architecture, consistently leads to superior prediction performance than DepInfeR, hence we recommend the data handling process above as it is more automated and requires less input from users.

### Posterior inference

In this section, we describe the posterior inference procedure of the proposed model using the modified kernel (9) and the likelihood functions given in (13). Let $p(f_{ps}|k_\nu ), p(\gamma ^2), p(\varvec{\alpha }|\gamma ^2), p(\sigma ^2|a_0,b_0), p(\varvec{Z}|\pi _0)$ be priors on the corresponding model parameters. Let $k_\nu (x_1,x_2)=\nu _1\exp \left( -\frac{(x_1-x_2)^2}{2\nu _2^2}\right)$ be the 1D Gaussian-RBF kernel with kernel parameter $\nu =\{\nu _1,\nu _2\}$, $\nu _1,\nu _2>0$. Under this choice of $k_\nu$, it is straightforward to verify that the modified kernel (9) takes the form $k_\nu ^{(0)}(x_1, x_2) = \nu _1\exp \left( -\frac{(x_1-x_2)^2}{2\nu _2^2}\right) - \nu _1\exp \left( -\frac{x_1^2 + x_2^2}{2\nu _2^2}\right)$. For the rest of the paper, we use this $k_\nu ^{(0)}(x_1, x_2)$ as the kernel function of the Gaussian process for simplicity. Simulation studies in the following sections show it achieves satisfactory results.

Let $J_{dp} =\mathbbm {1}(X_{dp} \text{ not } \text{ missing})$ be a binary variable indicating if $X_{dp}$ is missing. Let $\bar{\varvec{K}}^{(p)}$ be a $D\times D$ modified kernel matrix such that the entries $\bar{K}^{(p)}_{d_1d_2} = 0$ if $J_{d_1p}J_{d_2p} = 0$, and $\bar{K}^{(p)}_{d_1d_2} = k_\nu ^{(0)}(X_{d_1p}, X_{d_2p})$ otherwise for $d_1,d_2=1,\dots ,D$. Let $\varvec{1}^{D}$ be a $D \times D$ matrix with all entries being 1. Let $\varvec{0}_D$ be a zero vector of length *D*. Then by the conjugacy between Gaussian process priors on $f_{ps}$, Gaussian prior on $\alpha _s$, and the Gaussian likelihood on $\varvec{Y}_{obs}$, we can marginalize $\alpha _s$ and $\{f_{ps}\}_{p=1}^P$ out from (12), and obtain the following marginalized likelihood

14 $$\begin{aligned} p(\varvec{Y}_{-M_s}|\varvec{X}, \varvec{Z}, \sigma ^2, \gamma ^2, k_\nu ^{(0)} ) = \mathcal {N}\left( \varvec{Y}_{-M_s}; \varvec{0}_{D-|M_s|}, \bar{\Sigma }_s \right) \end{aligned}$$

where $$\bar{\Sigma }_s = \gamma ^2\varvec{1}^{D-|M_s|} + \sigma ^2 \varvec{I}_{D-|M_s|} + \sum _{p=1}^P z_p \bar{\varvec{K}}^{(p)}_{-M_s}$$ for $$s=1,\dots ,S$$.

Given the marginalized likelihood above, the posterior distribution of the set of model parameters $\{\varvec{Z}, \varvec{\alpha }, \{f_{ps}\}_{p,s=1}^{P,S}, \sigma ^2, \gamma ^2\}$ can then be factorized as

15 $$\begin{aligned}&p\left( \varvec{Z}, \varvec{\alpha }, \{f_{ps}\}_{p,s=1}^{P,S}, \sigma ^2, \gamma ^2|\varvec{X},\varvec{Y}_{obs}, k_\nu ^{(0)}, a_0, b_0, \pi _0\right) \nonumber \\ {}&\quad \propto \prod _{s=1}^S p(\alpha _s, \{f_{ps}\}_{p=1}^P|\sigma ^2, \gamma ^2, \varvec{X},\varvec{Y}_{-M_s}, \varvec{Z}, k^{(0)}_\nu ) \times p(\varvec{Z}, \gamma ^2, \sigma ^2| \varvec{X},\varvec{Y}_{obs}, k^{(0)}_\nu , a_0, b_0, \pi _0) \end{aligned}$$

where

16 $$\begin{aligned}&p(\alpha _s, \{f_{ps}\}_{p=1}^P|\sigma ^2, \gamma ^2, \varvec{X}, \varvec{Y}_{-M_s}, \varvec{Z}, k_\nu ^{(0)}) \nonumber \\&\quad \propto p\left( \varvec{Y}_{-M_s}|\varvec{X}, \alpha _s, \{f_{ps}\}_{p=1}^P, \varvec{Z}, \sigma ^2\right) p(\alpha _s|\gamma ^2) \prod _{p=1}^P p(f_{ps}|k_\nu ^{(0)}) \end{aligned}$$

for $$s=1,\ldots ,S$$, and

17 $$\begin{aligned}&p(\varvec{Z}, \gamma ^2, \sigma ^2| \varvec{X}, \varvec{Y}_{obs}, k_\nu ^{(0)}, a_0, b_0, \pi _0) \nonumber \\ {}&\quad \propto \prod _{s=1}^S p(\varvec{Y}_{-M_s}|\varvec{X}, \varvec{Z}, \sigma ^2, \gamma ^2, k_\nu ^{(0)} ) p(\gamma ^2)p(\sigma ^2|a_0,b_0)p(Z|\pi _0). \end{aligned}$$

This factorization suggests that we can approximately draw samples from the posterior by iteratively sampling from first $p(\alpha _s, \{f_{ps}\}_{p=1}^P|\sigma ^2, \gamma ^2, \varvec{X}, \varvec{Y}_{-M_s}, \varvec{Z}, k_\nu ^{(0)})$ for $s=1,..,S$ using Bayesian backfitting [10], and then the marginalized $p(\varvec{Z}, \gamma ^2, \sigma ^2| \varvec{X}, \varvec{Y}_{obs}, k_\nu ^{(0)}, a_0, b_0, \pi _0)$ using Metropolis-Hasting MCMC. Once we have obtained MCMC samples from the posterior, we are able to form both point and set estimates of the parameters of our interest such as $\text{ Pr }(z_p=1| \varvec{X}, \varvec{Y}_{obs}, k_\nu ^{(0)}, a_0,b_0,\pi _0)$, the posterior inclusion probability of the *p*th protein. Compared with DepInfeR, our Bayesian framework allows us to assess uncertainties in both the set of selected proteins and other model parameters in a straightforward fashion.

To demonstrate the efficacy of our method, we first apply the proposed model to a synthetic dataset (See Additional file 1: Sect. 3). Simulation results confirm that it can recover the underlying functions $f_{ps}$ accurately. From both simulation studies on synthetic and real (“GDSC1”, “BeatAML” and “EMBL” sections) datasets, we find that the fit of the proposed model is not sensitive to the choice of prior on $\gamma ^2$ or the choice of hyperparameters $\{a_0, b_0, \pi _0\}$, and primarily depends on the choice of kernel hyperparameter $\nu =\{\nu _1,\nu _2\}$. Therefore we recommend setting the prior on $\gamma ^2$ to be $\text{ Half-Normal }(0,1)$, $a_0=b_0=1$, $\pi _0=0.1$, and choosing $\nu$ using grid-search and 3-fold cross-validation.

## Outlines of simulation studies

In this section, we describe the setup of our numerical experiments. We will report the results in the next section.

### Data pre-processing

We use the same datasets analyzed in Batzilla et al. [3] to demonstrate the effectiveness of our proposed method. In addition to the original processed datasets used in DepInfeR, we also tried a different data pre-processing step in the following numerical experiments: For the drug-affinity matrix $\varvec{X}$, we denote $\varvec{X}_{\textrm{imp}}$ the original drug-affinity matrix used in DepInfeR [3] whose missing entries are filled in manually by the authors, and $\varvec{X}_{\textrm{miss}}$ the incomplete matrix without any imputation. For the drug-sensitivity matrix $\varvec{Y}$, we consider two choices: We denote $\varvec{Y}_{\textrm{imp}}$ the original imputed and *z*-score normalized drug-sensitivity matrix used in DepInfeR. We also construct a drug-sensitivity matrix from the incomplete raw sensitivity measures *without the imputation step*. The raw sensitivity measures in the GDSC1 dataset are all in the range (0, 1). We choose not to impute the missing values in the raw measures, and apply a data-independent logit transformation $h(y)=\log \frac{y}{1-y}$ (instead of the *z*-score normalization in DepInfeR) to map all non-missing raw sensitivity measures from (0, 1) to the real line. We denote $\varvec{Y}_{\textrm{logit}}$ the resulting logit-transformed incomplete drug-sensitivity matrix *without imputation*. For the beatAML and EMBL datasets in DepInfeR, we construct incomplete sensitivity matrices $\varvec{Y}_{\textrm{log}}$ in a similar fashion by applying data-independent log-transformation to the entries, mapping the positive scalar raw responses to the real line. In “GDSC1”, “BeatAML” and “EMBL” sections, we apply our proposed method to both the original sensitivity measures $\varvec{Y}_{\textrm{imp}}$ and our transformed, incomplete datasets $\varvec{Y}_{\textrm{logit}}$/$\varvec{Y}_{\textrm{log}}$, and demonstrate how such data-independent transformations lead to better prediction performance and normally distributed residuals, which agrees with our model assumption.

### Simulation strategy

We compare the prediction performance between the DepInfeR model based on the original dataset $\{\varvec{X}_{\textrm{imp}}, \varvec{Y}_{\textrm{imp}}\}$, and our proposed model based on two different datasets $\{\varvec{X}_{\textrm{miss}}, \varvec{Y}_{\textrm{imp}}\}$ (incomplete drug-protein affinity matrix and the original sensitivity matrix) and $\{\varvec{X}_{\textrm{miss}}, \varvec{Y}_{\textrm{logit}}\}$ or $\{\varvec{X}_{\textrm{miss}}, \varvec{Y}_{\log }\}$ (incomplete drug-protein affinity matrix and the incomplete, logit- or log-transformed sensitivity matrix). We include both the original and the incomplete drug-sensitivity matrices to demonstrate the effectiveness of the proposed data-independent transformation. Since $\varvec{Y}_{\textrm{imp}}$ and the transformed $\varvec{Y}_{\textrm{logit}}$ or $\varvec{Y}_{\textrm{log}}$ are not on the same scale, we compare the prediction performance between models with different datasets using *normalized* mean square error

$${\text{nMSE}}(\user2{Y},\widehat{\user2{Y}}) = \frac{{||\user2{Y} - \widehat{\user2{Y}}||_{2}^{2} }}{{||\user2{Y} - \user2{Y}|_{2}^{2} }}$$

as the accuracy benchmark, where $\hat{\varvec{Y}}$ is the estimate of the observed values $\varvec{Y}$ and $\bar{\varvec{Y}}$ is the sample mean of all entries in $\varvec{Y}$. Specifically, the estimated $\hat{\varvec{Y}}$ of our proposed model is computed as follows: For a fixed hyperparameter, we draw posterior samples of the parameters using the MCMC sampler described in “Posterior inference” section with chain length being fixed at 120. We discard the first 20 steps as burn-in, and retain the remaining 100 steps as our MCMC posterior samples. To illustrate that MCMC has converged in 120 iterations, we report the trace plots of the unnormalized log posterior density and $\sigma ^2$ of the proposed model fitted using the datasets $\{\varvec{X}_{\textrm{miss}}, \varvec{Y}_{\textrm{logit}}\}$ or $\{\varvec{X}_{\textrm{miss}}, \varvec{Y}_{\textrm{log}}\}$. For each dataset in Batzilla et al. [3], we run 6 MCMC with random initializations and see no evidence of poor mixing from the trace plots. In addition, the Gelman-Rubin statistics of the scalar parameter $\sigma ^2$ and $\gamma ^2$ are both less than 1.1, indicating good convergence (see Additional file 1: Sect. 1.2 for details). On average, each repetition of posterior inference using the MCMC described in “Posterior inference” section takes $2 \sim 2.5$ hours to run on our machine. For $i=1,\dots ,100$, we then compute the estimated responses $\hat{\varvec{Y}}_i$ based on the *i*th MCMC sample as the model parameters. We then report the sample average $\hat{\varvec{Y}}=\frac{1}{100}\sum _{i=1}^{100}\hat{\varvec{Y}}_i$ as our final estimated responses of $\varvec{Y}$. The normalized MSE measures the prediction error of a model relative to the variability of the dataset, hence allows us to compare the performance of models fitted using datasets that have different value ranges. Similar to Batzilla et al. [3], the $\textrm{nMSE}$ of the model under different hyper-parameters are estimated using 3-fold cross validation, and the optimal hyper-parameters are chosen using grid-search.

In addition to DepInfeR and our proposed method, we also tried to regress $\varvec{Y}_{\textrm{imp}}$ on $\varvec{X}_{\textrm{imp}}$ (i.e. the original dataset) using Multivariate Random Forest [11, 23], a non-additive, non-linear multivariate regression model, and report its prediction performance. We choose to include this highly flexible model as a benchmark of prediction accuracy since we would like to check to what degree does the additive structure in both DepInfeR and our proposed method affects prediction power.

### Protein selection

In addition to prediction performance, we also compare the set of proteins (kinases in the following examples) selected by DepInfeR and our proposed model. For our approach, we record the proteins whose corresponding indicator $z_p$ is 1 for more than $95\%$ of the times in the MCMC samples, and treat them as the set of selected proteins. For each dataset and each choice of hyper-parameter, we report the Intersection over Union $\textrm{IoU}(A,B)=\frac{|A\cap B|}{|A \cup B|}$ as a similarity measure between the subsets of proteins selected by our approaches and the ones reported in DepInfeR. For each dataset, we also report the subsets of proteins selected by the proposed model that attain minimal cross-validation error (Fig. 4).

## Results

In this section, we demonstrate the efficacy of our proposed method using the same datasets analyzed in Batzilla et al. [3].

**Fig. 2:**
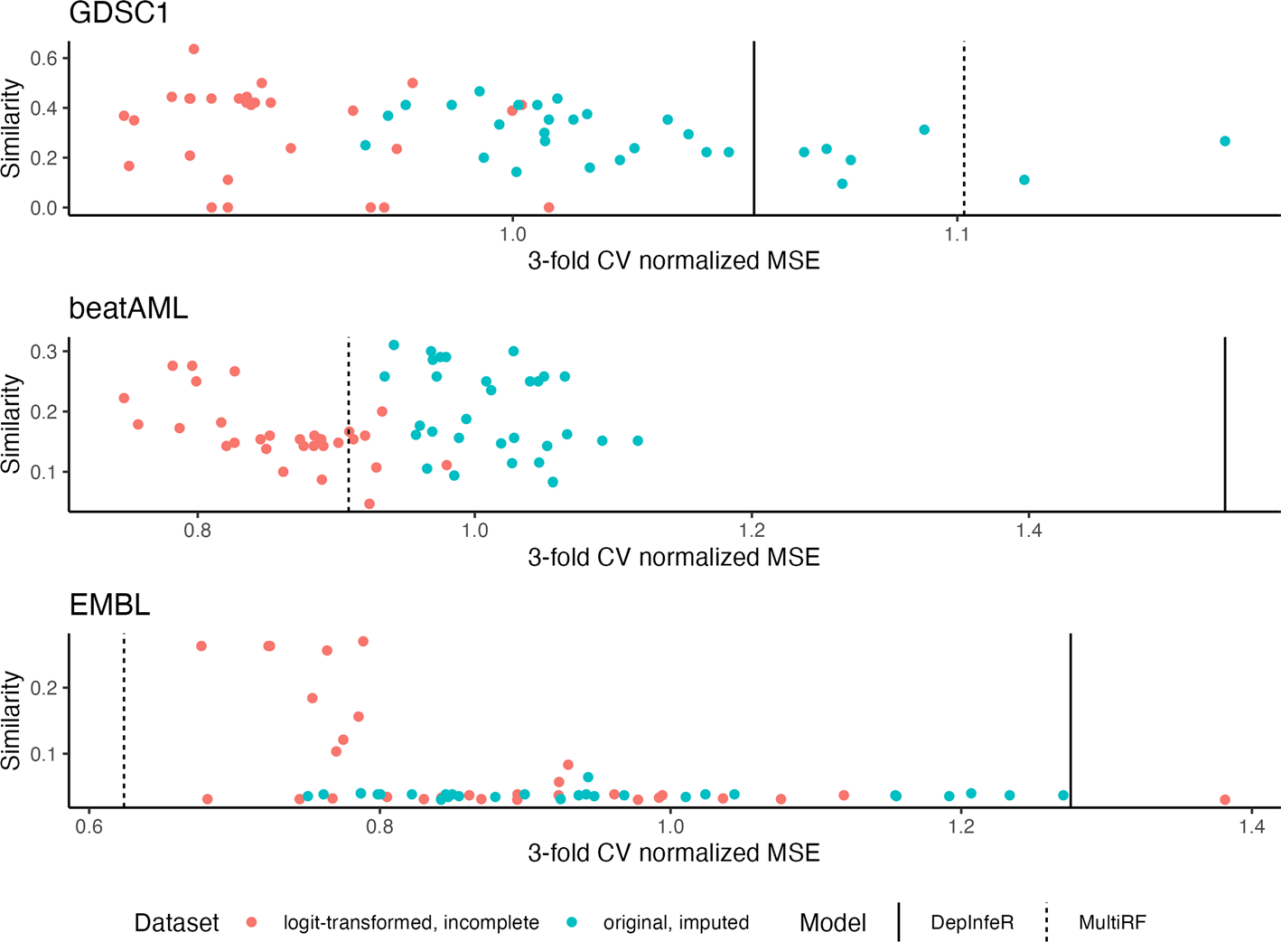
Prediction performance: Each point in each figure corresponds to a model fitted under a given value of hyper-parameter using either the original, imputed $\varvec{Y}_{\textrm{imp}}$ or the incomplete, logit-(log-)transformed $\varvec{Y}_{\textrm{logit}}$($\varvec{Y}_{\textrm{log}}$). The horizontal coordinate of the point is the normalized MSE of the model estimated using 3-fold CV, and the vertical coordinate is the Intersection-over-Union score between the subset of kinases selected by the fitted model and the corresponding subset of kinases selected by DepInfeR. The vertical dashed and solid lines correspond to the estimated normalized MSE of multivariate Random Forest and DepInfeR based on the original dataset $\{\varvec{X}_{\textrm{imp}}, \varvec{Y}_{\textrm{imp}}\}$

### GDSC1

In this section we compare the performance of our proposed method with DepInfeR using the GDSC1 dataset studied in Batzilla et al. [3]. The GDSC1 dataset consists of tumor specimens from different cancer types: 30 samples from diffuse large B-cell lymphoma (DLBCL) patients, 25 samples from acute lymphocytic leukemia (ALL) patients, 24 samples from acute myeloid leukemia (AML) patients and 47 samples from breast carcinoma (BRCA) patients. We run our proposed model multiple times under different choices of the hyper-parameter $\nu =\{\nu _1, \nu _2\}$. Specifically, we consider $\nu _1\in \{0.01, 0.0825, 0.155, 0.2275, 0.3000\}$, $\nu _2 \in \{0.01, 0.068, 0.126, 0.184, 0.242,0.3\}$ and tried all their combinations, resulting in 30 distinct hyper-parameter values in a grid. We then estimate the normalized MSE of the fitted model under each choice of hyper-parameter using 3-fold CV. From Fig. 2 we see that under both $\{\varvec{X}_{\textrm{miss}}, \varvec{Y}_{\textrm{imp}}\}$ and $\{\varvec{X}_{\textrm{miss}}, \varvec{Y}_{\textrm{logit}}\}$, our proposed approach outperforms DepInfeR (solid vertical black line) for all choices of hyper-parameters, and outperforms the flexible MultiRF model for most of the times. In addition, we see the choice of hyperparameter $\nu$ has impact on both prediction accuracy and the subsets of selected kinases.

**Fig. 3:**
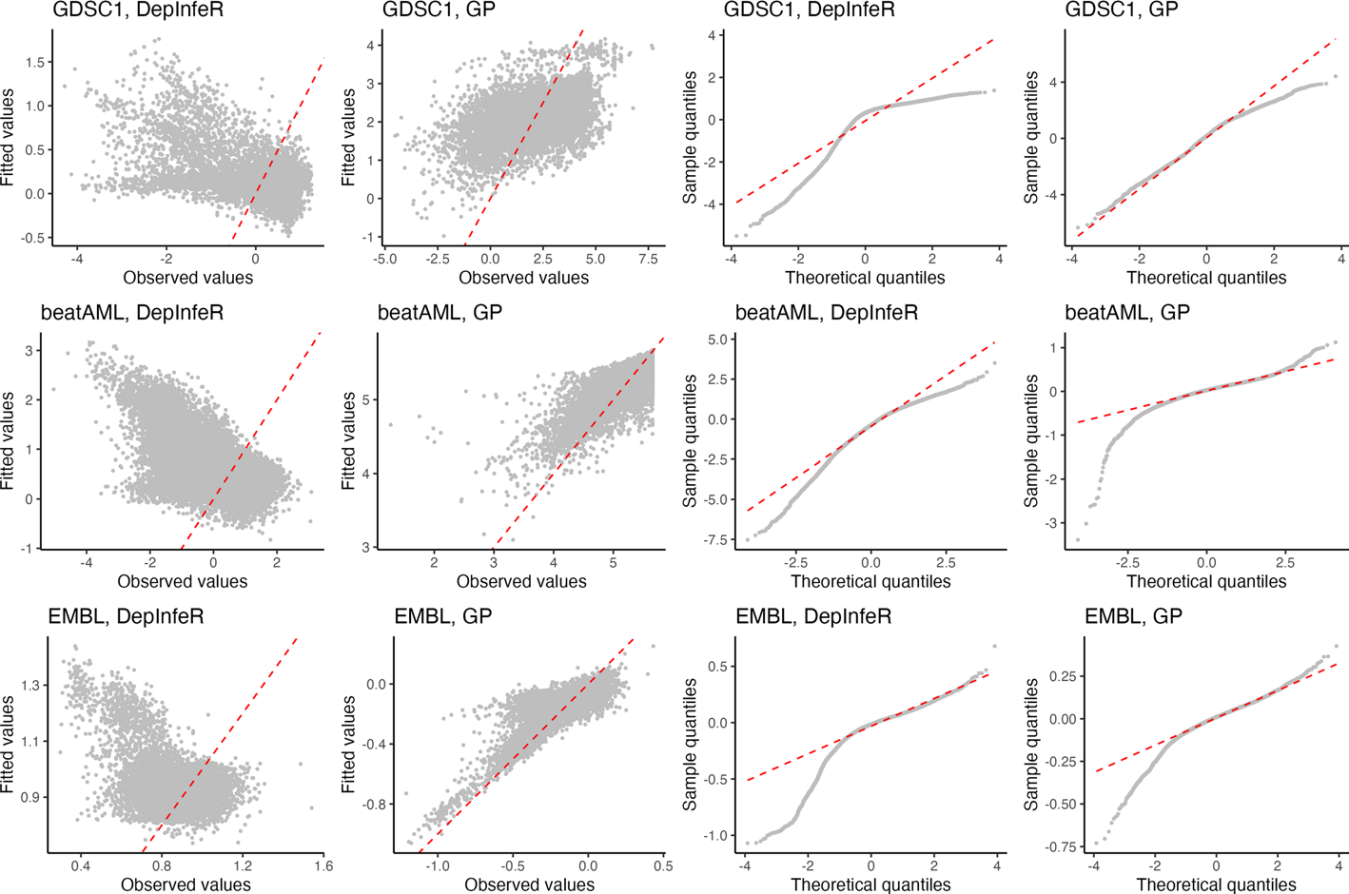
Observed vs estimated responses plots and residual Q-Q plots for both DepInfeR and the proposed method with different datasets. From left to right: Observed vs estimated responses plot of our proposed model; Observed vs estimated responses plot of DepInfeR; Residual Q-Q plot of our proposed model; Residual Q-Q plot of DepInfeR. Note that the scale of the datasets used to fit our proposed model are different from the ones used to fit DepInfeR

We also see that the model with $\varvec{Y}_{\textrm{logit}}$ tend to achieve lower normalized MSE than the one with $\varvec{Y}_{\textrm{imp}}$, which indicates the efficacy of the logit transformation. To further compare the logit and the original z-score transformations, we report the residual Q-Q plot and the observed vs fitted responses plot for both DepInfeR with the original $\{\varvec{X}_{\textrm{imp}}, \varvec{Y}_{\textrm{imp}}\}$ and the proposed method with the new $\{\varvec{X}_{\textrm{miss}}, \varvec{Y}_{\textrm{logit}}\}$ under the hyperparameters that lead to minimal normalized MSE in Fig. 3. We see that the proposed method with the new dataset fits the observed responses better, and its residuals are roughly normally distributed, indicating that the fitted results are in-line with our model assumption. This further supports that our proposed model with the data-independent logit transformation leads to better prediction performance.

**Fig. 4:**
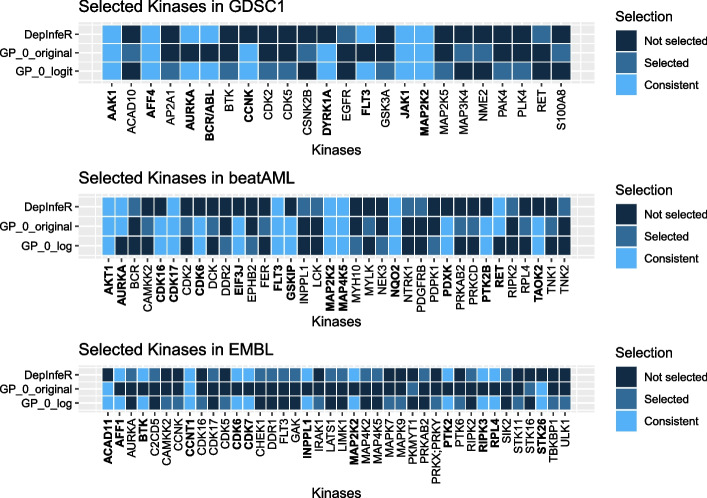
Feature Selection Comparison: Sets of kinases reported in Batzilla et al. [3] and the ones selected by our proposed model under the hyperparameters $\nu ^*$ that lead to the minimal normalized MSE. We highlight the kinases that appear in more than one of the reported subsets. Top: Selected kinases in the GDSC1 dataset. Compared with DepInfeR, the proposed models also suggest that CCNK and DYRK1A are informative to the sensitivity measure. Mid: Selected kinases in the EMBL dataset. The proposed models also suggest that CDK16, CDK6, EIF3J, GSKIP, PDXK, PTK2B and TAOK2 are also informative. Bottom: Selected kinases in the EMBL dataset. he proposed models also suggest that ACAD11 and STK26 are informative

**Fig. 5:**
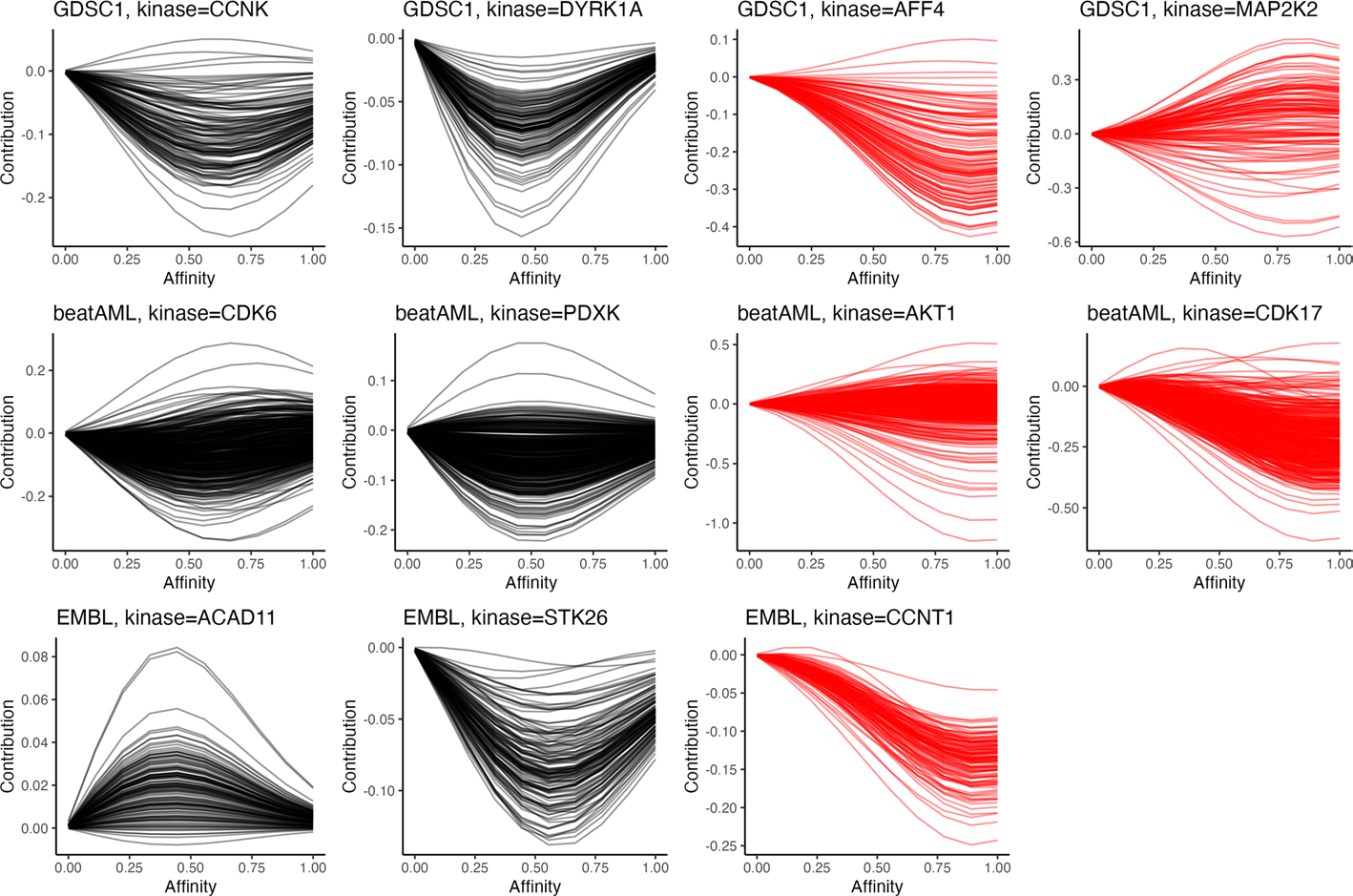
Estimating $f_{ps}$: Estimated posterior means of $f_{ps}$ of a single kinase *p* across all sample cells $s=1,\ldots ,S$ based on the logit- or log-transformed incomplete responses. First two columns: Examples of estimated $f_{ps}$ of kinases that are not selected by DepInfeR. Last two columns: Examples of kinases that are selected by both DepInfeR and our approach. Note that our estimated $f_{ps}$ are not directly comparable with DepInfeR as they are fitted using responses matrices on different scales

We also would like to highlight that even though a number of hyper-parameters are able to attain a similar level of prediction accuracy in Fig. 2, their corresponding similarity measures between the subsets of kinases selected by the fitted model and DepInfeR vary considerably. This suggests that there may exist many subsets of kinases that are equally informative to the drug-sensitivity measures. In Fig. 4 we report the subsets of kinases selected by the proposed model with the choice of hyperparameter that attains minimal normalized MSE under the two choices of datasets. We see DYRK1A and CCNK are not selected by the original DepInfeR but are consistently picked up by our proposed model. Experimental studies confirm that DYRK1A is associated with acute lymphocytic leukemia (ALL) [4], acute myeloid leukemia (AML) [15] and breast cancer (BRCA) [14]. On the other hand, CCNK is complexed with kinase CDK12 and CDK13 [5, 9], which are strongly associated with BRCA [13], AML [21] and diffuse large B-cell lymphoma (DLBCL) [8]. Demonstrated that the targeted degradation of CCNK/CDK12 complex is a druggable vulnerability of colorectal cancer [7] . This finding suggests that there might be similar druggable dependencies between CCNK/CDK12 or CCNK/CDK13 complexes and the cancer types included in the GDSC1 dataset such as BRCA and AML.

To better understand this difference in kinase selection, we also report the estimated posterior means of $f_{ps}$ of CCNK and DYRK1A across all sample cells. From Fig. 5 we see the estimated $f_{ps}$ have a non-monotonic $\cap$- or $\cup$-shape, which can not be well approximated by the linear basis function in DepInfeR. In contrast, the $f_{ps}$ of kinases that are selected by both DepInfeR and our proposed method are indeed more monotonic, allowing the linear basis functions in DepInfeR to approximate these patterns reasonably well. This confirms that in addition to monotonic or linear dependencies, our proposed approach are also able to capture non-linear dependencies that can not be identified by DepInfeR.

### BeatAML

In this section, we compare the performance of our proposed approach with DepInfeR using the beatAML dataset in Batzilla et al. [3]. The beatAML dataset consists of tumor specimens collected from 528 AML patients. We fit our proposed models using the two datasets $\{\varvec{X}_{\textrm{miss}}, \varvec{Y}_{\textrm{imp}}\}$ and $\{\varvec{X}_{\textrm{miss}}, \varvec{Y}_{\textrm{log}}\}$, and estimate the normalized MSE repeatedly using the same grid of hyper-parameters and procedure described in “GDSC1” section.

From Fig. 2 we see our fitted model under both response matrices ($\varvec{Y}_{\textrm{log}}, \varvec{Y}_{\textrm{imp}}$) outperforms DepInfeR (solid vertical black line) in term of prediction accuracy for all choices of hyper-parameters, and our proposed model fitted to the new dataset $\{\varvec{X}_{\textrm{miss}}, \varvec{Y}_{\textrm{log}}\}$ is able to outperform the highly flexible MultiRF. We also report the residual Q-Q plot and the observed vs fitted responses for both DepInfeR with $\{\varvec{X}_{\textrm{imp}}, \varvec{Y}_{\textrm{imp}}\}$ and the proposed method with $\{\varvec{X}_{\textrm{miss}}, \varvec{Y}_{\textrm{log}}\}$ in a similar fashion to the previous section. Again from Fig. 3 we also see our proposed model with the log-transformed sensitivity measure has better model fit, and its residuals are not drastically different from a normal distribution.

Figure 4 shows that CDK16, CDK6, EIF3J, GSKIP, PDXK, PTK2B and TAOK2 are not selected by DepInfeR but are consistently picked up by our model. Various experimental studies confirm association between AML and CDK6 [26], GSKIP [18, 19], PDXK [6] and PTK2B [1, 27] also suggests a possible indirect association between AML and TAOK2.

Similar to the previous section, we also report the estimated posterior means of $f_{ps}$ of CDK6 and PDXK across all sample cells. From Fig. 5 we see the estimated $f_{ps}$ also have a non-monotonic $\cap$- or $\cup$-shape, while the $f_{ps}$ of kinase AKT1 and CDK17, which are selected by both DepInfeR and our proposed method, are more monotonic.

### EMBL

In this section, we compare the performance of our proposed approach with DepInfeR using the EMBL dataset in Batzilla et al. [3]. The EMBL dataset consists of 117 tumor samples from CLL patients, 7 samples from mantle cell lymphoma (MCL) patients, and 7 samples from T-cell prolymphocytic leukemia (T-PLL) patients. Here we run the proposed model and estimate the normalized MSE using exactly the same setup as in “BeatAML” section. From Fig. 2 we see that our proposed approach outperforms DepInfeR consistently (solid vertical black line) for all choices of hyper-parameters. The proposed model with the log-transfromed, incomplete sensitivity measure $\varvec{Y}_{\textrm{log}}$ tend to achieve better performance than the model with the original, imputed sensitivity measure $\varvec{Y}_{\textrm{imp}}$, and is able to attain a comparable prediction performance to the more flexible MultiRF model while maintain interpretability. We also report the residual Q-Q plot and the observed vs fitted responses plot for both DepInfeR with $\{\varvec{X}_{\textrm{imp}}, \varvec{Y}_{\textrm{imp}}\}$ and the proposed method with $\{\varvec{X}_{\textrm{miss}}, \varvec{Y}_{\textrm{log}}\}$ in a similar fashion to the previous section in Fig. 3. Here we also see that our proposed approach with the new dataset achieves better model fit, and the residuals are also reasonable close to a normal distribution.

From Fig. 4 we see ACAD11 and STK26 are not selected by the original DepInfeR but are consistently chosen by our model. The EMBL dataset primarily consists of tumor samples from chronic lymphocytic leukemia (CLL) patients. To our best knowledge, direct association between ACAD11 and CLL has not been reported before. However, [12] show that ACAD11 plays a key role in the pro-survival function of p53 tumor suppressor, a strong molecular predictors for CLL [29]. On the other hand, [17] recommends targeting p53 and restoring the function of the disrupted p53 pathway in the treatment of CLL. This suggests the potential therapeutic value of ACAD11 in CLL treatment. Although the association between kinase STK26 and CLL is unclear, its association with AML is recently reported in [25]. This finding suggests possible dependency between STK26 and CLL, further indicating the clinical potential of our method.

Similar to the previous section, we compare the estimated $f_{ps}$ between kinases not selected by DepInfeR (ACAD11 and STK26) and the one selected by both DepInfeR and our method (CCNT1). Figure 5 shows that the shape of the estimated $f_{ps}$ follow patterns similar to the ones demonstrated in previous sections.

## Discussion

In this paper, we proposed a Bayesian extension of DepInfeR [3], a computational framework for identifying the sample-specific protein dependencies (i.e. to what extent does the survival of the cancer cells depend on a certain protein) using both the drug-sensitivity and drug-protein affinity data. Compared with DepInfeR, our proposed approach uses Gaussian process to model the unobserved dependency structures between proteins and cell samples, and uses Spike-and-Slab prior to decouple protein selection and parameter regularization. This modelling framework allows users to identify non-linear protein-cancer cell dependencies, and make probabilistic statements regrading the inclusion of candidate proteins. In addition, our method does not any require any imputation on either the drug-sensitivity or the drug-protein affinity data. As a result, our approach requires less input from the users, and is more automated than DepInfeR.

In simulation studies, we demonstrated that our approach consistently outperformed DepInfeR in term of prediction accuracy, and was able to identify known protein-cancer cell dependencies that were not picked up by DepInfeR [3]. In addition, our approach also detected a number of protein-cancer cell dependencies that have not been reported in literature. These findings support the therapeutic potential of the proposed method, and confirm that our proposed methods can help revealing more insights into protein-cancer cell dependencies, and finding new possibilities for patient or cancer type specific pharmacological intervention.

### Supplementary Information

**Additional file 1.** Supplementary information providing further simulation results, derivation and implementation of a linear version of the model and additional synthetic data experiments.

## Data Availability

All data and code used in this study are in the previously published repository: https://github.com/Huber-group-EMBL/DepInfeR_workflow.

## Code availability

An R implementation of the proposed method and the numerical experiments is available at https://github.com/hwxing3259/depinfer_gp.
