# Supplementary material for: Bayesian inference for identifying tumour-specific cancer dependencies through integration of ex-vivo drug response assays and drug-protein profiling
Source: BMC Bioinformatics. 2024 Mar 8;25:104. doi: 10.1186/s12859-024-05682-0 (PMC10921766; doi:10.1186/s12859-024-05682-0)
Supplement: Supplementary file 1 — Additional file 1. Supplementary information providing further simulation results, derivation and implementation of a linear version of the model and additional synthetic data experiments. [file 12859_2024_5682_MOESM1_ESM.pdf]

Supplementary Information

Hanwen Xing[1*] and Christopher Yau[1,2]

[1*]Nuffield Department of Women's and Reproductive Health, University of Oxford, Oxford, UK.
[2]Health Data Research UK, London, UK.

*Corresponding author(s). E-mail(s): hanwen.xing@wrh.ox.ac.uk;
Contributing authors: christopher.yau@hdruk.ac.uk;

Contents

1 Additional Simulation results **3**
- 1.1 The elementwise median in DepInfeR . . . 3
- 1.2 MCMC convergence of the fitted models . . . 3
- 1.3 Assumption of independent residuals . . . 4

2 Spike-and-slab linear regression **5**
- 2.1 Missing values in X, Y . . . 5
- 2.2 Posterior inference . . . 6
- 2.3 L_2 regularization on regression parameter β_s . . . 7

3 A Synthetic example **8**

4 Prediction performance of the linear model **9**

List of Figures

1 How taking elementwise median affects DepInfeR's model fit: Left-most column contains the observed vs fitted responses plots of DepInfeR for the three datasets respectively. The rest of each row contains the observed vs fitted responses plots of repeated runs of a single MultiLASSO regression model fitted using the corresponding dataset. 3

2 Trace plots of the unnormalized log posterior density and σ^2 of the proposed model based on 6 repeated runs with GDSC1 dataset $\{X_{miss}, Y_{logit}\}$ and random initialization. 10
3 Trace plots of the unnormalized log posterior density and σ^2 of the proposed model based on 6 repeated runs with beatAML dataset $\{X_{miss}, Y_{log}\}$ and random initialization. 11
4 Trace plots of the unnormalized log posterior density and σ^2 of the proposed model based on 6 repeated runs with EMBL dataset $\{X_{miss}, Y_{log}\}$ and random initialization. 12
5 Left: Heatmaps of the correlation matrices, each entry in the correlation matrix corresponds to the correlation between the fitted residual vectors of two drugs. Right: Histogram of the below-diagonal entries of the correlation matrix on the left. 13
6 **A**: The proposed Spike-and-Slab linear regression model in matrix form. Here we assume $D = 2,\ S = 3,\ P = 4$. **B**: Graphical representation of the proposed Spike-and-Slab linear regression model. **C**: A table of all variables used to define the proposed model. 14
7 **Synthetic Data Experiment.** Plots of the basis functions $\{f_i\}_{i=1}^4$. 14
8 f_{sp} for $p = 1,\ s = 1, \ldots, 9$ estimated based on the full dataset without any missing values. The shaded regions correspond to the 95% credible band of the estimated mean functions. 15
9 f_{sp} for $p = 1,\ s = 1, \ldots, 9$ estimated based on the dataset with missing level 30%. The shaded regions correspond to the 95% credible band of the estimated mean functions. 16
10 f_{sp} for $p = 1,\ s = 1, \ldots, 9$ estimated based on the dataset with missing level 30%. The shaded regions correspond to the 95% credible band of the estimated mean functions. 17
11 f_{sp} for $p = 1,\ s = 1, \ldots, 9$ estimated based on the dataset with missing level 30%. The shaded regions correspond to the 95% credible band of the estimated mean functions. 18
12 **GDSC1** results. Each point corresponds to a model fitted under a given value of hyper-parameter. The horizontal coordinate of the point is the normalized MSE of the model estimated using 3-fold CV, and the vertical coordinate is the Intersection-over-Union score between the set of protein selected by the fitted model and the set of protein selected by DepInfeR. The vertical dashed and solid black lines correspond to the estimated normalized MSE of multivariate Random Forest and DepInfeR based on the original dataset $\{X_{imp}, Y_{imp}\}$. 19
13 **beatAML results**. Lines and points have the same interpretation as in Fig 12. 20
14 **EMBL results**. Lines and points have the same interpretation as in Fig 12 21

List of Tables

1 Gelman-Rubin statistics $\hat{R}$ of σ^2 and γ^2 on the three datasets we studied. The upper 95% C.I. of the $\hat{R}$ statistics are reported in brackets. 4

1 Additional Simulation results

1.1 The elementwise median in DepInfeR

Here we examine how taking the elementwise median affect the fitting of DepInfeR. In Fig 1, we report the observed vs estimated responses plot of DepInfeR for each of the datasets considered in [1]. For each of the dataset, we also repeatedly fit a single multivariate-LASSO model, and report the observed vs estimated responses plot for each repetition. We see even though taking elementwise median improves the robustness of DepInfeR, it does affect the fitting of the model.

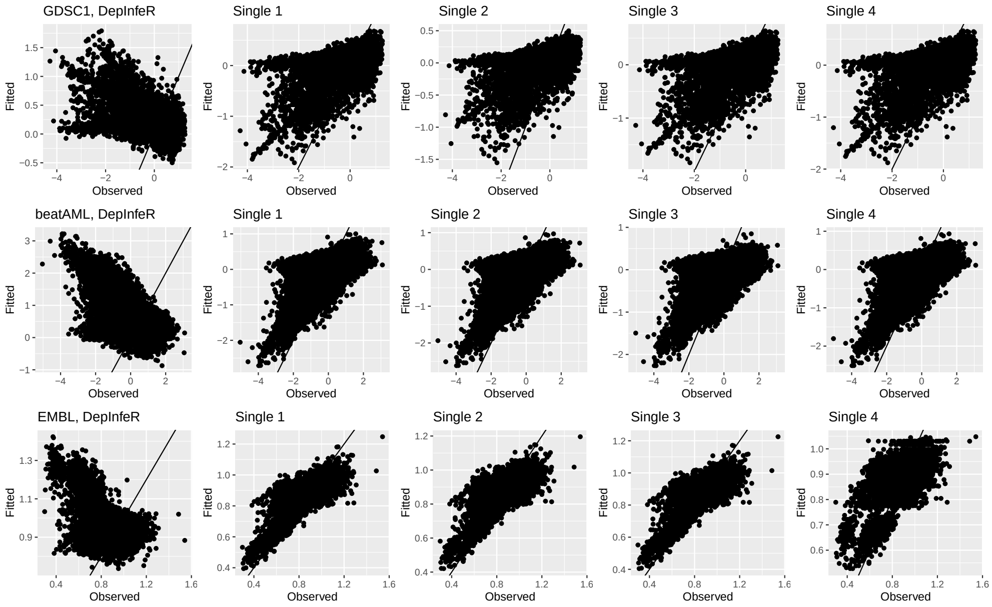

Fig. 1 How taking elementwise median affects DepInfeR's model fit: Left-most column contains the observed vs fitted responses plots of DepInfeR for the three datasets respectively. The rest of each row contains the observed vs fitted responses plots of repeated runs of a single MultiLASSO regression model fitted using the corresponding dataset.

1.2 MCMC convergence of the fitted models

In this section we report the trace plots of the log posterior density and the scalar parameter σ^2 of the MCMC outputs from the GDSC1, BeatAML and EMBL experiments in the main manuscript. From Fig 2, 3 and 4 we see no evidence of poor mixing. We also report the Gelman-Rubin statistics for parameter σ^2 and γ^2 in Table 1. Both the trace plots and the Gelman-Rubin statistics look healthy, hence we believe our MCMC algorithms have converged.

	σ^2	γ^2
GDSC1	1.017(1.050)	0.999(1.012)
beatAML	1.007(1.030)	1.019(1.060)
EMBL	1.009(1.030)	0.998(1.010)

Table 1 Gelman-Rubin statistics $\hat{R}$ of σ^2 and γ^2 on the three datasets we studied. The upper 95% C.I. of the $\hat{R}$ statistics are reported in brackets.

1.3 Assumption of independent residuals

Our proposed model assumes that the residuals ϵ_{ds} are i.i.d. Gaussian. This assumption of independent residuals is adopted from DepInfeR: The group-LASSO model used in DepInfeR is fitted by minimizing a penalized L_2 loss on the residuals, which is equivalent to maximizing a penalized i.i.d. Gaussian likelihood of the residuals. The correlated residuals would improve the flexibility of the model and reveal finer interaction between the drugs and the samples. However, allowing the residuals to be correlated would introduce a new set of parameters that scale quadratically with the size of the dataset, and make posterior inference more challenging and computationally demanding (even though it is straightforward to write down the corresponding likelihoods and unnormalized posterior densities). Compared with correlated residuals, the independent assumption also makes prediction simpler: If we assume the residuals are correlated, then whenever a new drug is presented, users have to additionally estimate the correlation between this new drug to the existing ones in order to get the predicted sensitivity measure. In contrast, our proposed model with independent residuals can predict new drug-sensitivity measure in a plug-in fashion. For each of the dataset we studied in the paper, we also report the pairwise correlation between the fitted residual vectors of different drugs in the dataset (each residual vector associated with a drug d has entries $\hat{\epsilon}_{ds} = Y_{ds} - \hat{Y}_{ds}$ for $s = 1, ..., S$) in Fig 5. We see the correlations are centered at 0, and the magnitude of the majority of the correlations are less than 0.3. Hence we conclude that there is no evidence of strong correlation between drugs in the datasets we studied in this paper.

2 Spike-and-slab linear regression

In the main text, we proposed a flexible regression model with Gaussian process priors. It is also possible to follow the linear assumption in DepInfeR and construct a spike and slab linear regression model [3, 5].

Let $\pi_0 \in (0,1)$, $a_0, b_0, c_0 \in \mathbb{R}^+$ be hyperparameters. We define the linear spike-and-slab model using the following generative process:

$$z_p \sim \text{Bernoulli}(\pi_0),\ p = 1, ..., P; \tag{1}$$

$$\sigma^2 \sim \text{Inv-Gamma}(a_0, b_0); \tag{2}$$

$$\alpha_s|\sigma^2 \sim \mathcal{N}\left(0, c_0^{-1}\sigma^2\right),\ s = 1, ..., S; \tag{3}$$

$$\beta_s|\sigma^2 \sim \mathcal{N}\left(\mathbf{0}_P, c_0^{-1}\sigma^2 I_P\right),\ s = 1, ..., S; \tag{4}$$

$$\epsilon_{ds} \sim \mathcal{N}(0, \sigma^2),\ d = 1, ..., D, s = 1, ..., S; \tag{5}$$

$$Y_{ds} = \alpha_s + \sum_{p=1}^{P} z_p X_{dp} \beta_{ps} + \epsilon_{ds},\ d = 1, ..., D, s = 1, ..., S, \tag{6}$$

where $\mathbf{0}_P$ is a P dimensional zero vector, I_P is the $P \times P$ identity matrix, Y_s is the sth column of Y, and $Z = \{z_p\}_{p=1}^P$. Here the binary variables $\{z_p\}_{p=1}^P$ indicates if the pth protein contributes to the sensitivity measure for $p = 1, ..., P$, σ^2 controls the scale of the noise in the drug-sensitivity matrix Y, and $\alpha_s \in \mathbb{R}$, $\beta_s \in \mathbb{R}^P$ are the regression coefficients for $s = 1, ..., S$. See Fig 6 for a schematic illustration.

2.1 Missing values in $\boldsymbol{X}, \boldsymbol{Y}$

We can marginalize out the missing entries in Y, and carry out posterior inference solely based on the observed Y_{obs}. Under the linear spike-and-slab model, the observed Y_{-M_s} follows

$$p(Y_{-M_s}|X, Z, \alpha_s, \beta_s, \sigma^2) = \mathcal{N}(Y_{-M_s}; \alpha_s \mathbf{1}_{D-|M_s|} + X_{-M_s}(Z * \beta_s), \sigma^2 I_{D-|M_s|}), \tag{7}$$

where X_{-M_s} is the processed drug-affinity matrix X with rows whose indices are in M_s being removed, $Z * \beta_s$ represents element-wise multiplication, $\mathbf{1}_{D-|M_s|}$ is a $D - |M_s|$ dimensional vector with all entries being 1, and $|M_s|$ is the size of the set M_s (i.e. number of missing values in Y_s). Let $Y_{obs} = \{Y_{-M_s}\}_{s=1}^S$ be the collection of observed entries in Y. Let $\alpha = \{\alpha_s\}_{s=1}^S$, $\beta = \{\beta_s\}_{s=1}^S$ be the sets of regression parameters. The likelihood function of the observed values Y_{obs} then can be written as

$$p(Y_{obs}|X, Z, \alpha, \beta, \sigma^2) = \prod_{s=1}^{S} \mathcal{N}(Y_{-M_s}; \alpha_s \mathbf{1}_{D-|M_s|} + X_{-M_s}(Z * \beta_s), \sigma^2 I_{D-|M_s|}), \tag{8}$$

where $\mathcal{N}(\cdot; \mu, \Sigma)$ is the multivariate Gaussian density with mean μ and covariance matrix Σ.

In addition to missing values in the drug-sensitivity matrix Y, missing entries in the drug-affinity matrix X are also handled in a same fashion as in Sec **??**. As a result, each sensitivity measure Y_{ds} under the linear model can be written as

$$Y_{ds} = \alpha_s + \sum_{p=1}^{P} \mathbb{1}(X_{dp} \text{ not missing}) z_p X_{dp} \beta_{ps} + \epsilon_{ds}. \tag{9}$$

For our linear model, it is easy to verify that handling missing values in X using indicator variables as above is equivalent to filling all missing entries in the process drug-affinity matrix X by 0.

2.2 Posterior inference

In this section, we describe the posterior inference procedure of the linear spike-and-slab model. Let $p(\alpha, \beta, \sigma^2 | a_0, b_0, c_0)$ and $p(Z|\pi_0)$ be the prior distributions on α, β, σ^2 and Z respectively. The posterior distribution of the inclusion indicators Z, the regression coefficients α, β and the variance σ^2 can be written as

$$p(Z, \alpha, \beta, \sigma^2 | X, Y_{obs}, a_0, b_0, c_0, \pi_0) \propto \\ p(Y_{obs}|X, Z, \beta, \sigma^2) p(Z|\pi_0) p(\alpha, \beta, \sigma^2 | a_0, b_0, c_0). \tag{10}$$

Because of the conjugacy between $p(Y_{obs}|X, Z, \alpha, \beta, \sigma^2)$ and $p(\alpha, \beta, \sigma^2 | a_0, b_0, c_0)$, we are able to sample from the posterior $p(Z, \alpha, \beta, \sigma^2 | X, Y_{obs}, a_0, b_0, c_0, \pi_0)$ using a collapsed Gibbs sampler, which greatly improves the efficiency of posterior inference. Let $\text{diag}(Z)$ be a $P \times P$ diagonal matrix with diagonal elements being entries in Z and off-diagonal elements being 0. Let $\bar{X} = (\mathbf{1}_P, X)\,\text{diag}(Z)$ (i.e. appending a column vector of 1 to the X matrix, then multiply by $\text{diag}(Z)$). Let

$$\mu_s = \left(\bar{X}_{-M_s}^T \bar{X}_{-M_s} + c_0 I_{D-|M_s|}\right)^{-1} \bar{X}_{-M_s}^T Y_{-M_s}, \; s = 1, ..., S; \tag{11}$$

$$\Sigma_s = \sigma^2 \left(\bar{X}_{-M_s}^T \bar{X}_{-M_s} + c_0 I_{D-|M_s|}\right)^{-1}, \; s = 1, ..., S; \tag{12}$$

$$a_n = a_0 + \frac{DS - \sum_{s=1}^{S} |M_s|}{2}; \tag{13}$$

$$b_n = b_0 + \frac{1}{2} \sum_{s=1}^{S} Y_{-M_s}^T \left(I_{D-|M_s|} + c_0^{-1} \bar{X}_{-M_s} \bar{X}_{-M_s}^T\right)^{-1} Y_{-M_s}. \tag{14}$$

We can then factorize the posterior density as

$$p(Z, \alpha, \beta, \sigma^2 | X, Y_{obs}, a_0, b_0, c_0, \pi_0) \propto \\ \prod_{s=1}^{S} p(a_s, \beta_s | X, Y_{-M_s}, \sigma^2, c_0) p(\sigma^2 | X, Y_{obs}, Z, a_0, b_0) \times p(Z | X, Y_{obs}, a_0, b_0, c_0, \pi_0), \tag{15}$$

where

$$p(a_s, \beta_s | X, Y_{-M_s}, \sigma^2, c_0) = \mathcal{N}\left(\{\alpha_s, \beta_s\}; \mu_s, \Sigma_s\right), \ s = 1, ..., S, \tag{16}$$

$$p(\sigma^2 | X, Y_{obs}, Z, a_0, b_0) = \text{Inv-Gamma}\left(\sigma^2; a_n, b_n\right) \tag{17}$$

and

$$p(Z|X, Y_{obs}, a_0, b_0, c_0, \pi_0) \propto \frac{\Gamma(a_n)}{b_n^{a_n}} \prod_{s=1}^{S} \det\left(I_{D-|M_s|} + c_0^{-1} \bar{X}_{-M_s} \bar{X}_{-M_s}^T\right)^{-\frac{1}{2}} p(Z|\pi_0). \tag{18}$$

We use a collapsed Gibbs sampler [2, 4] to draw posterior samples from $p(Z, \alpha, \beta, \sigma^2 | X, Y_{obs}, a_0, b_0, c_0, \pi_0)$ by iteratively sampling from $p(\sigma^2 | X, Y_{obs}, Z, a_0, b_0)$ and $p(a_s, \beta_s | X, Y_{-M_s}, \sigma^2, c_0)$ for $s = 1, ..., S$ directly, and then $p(Z|X, Y_{obs}, a_0, b_0, c_0, \pi_0)$ using Metropolis-Hasting MCMC. We run additional simulation studies comparing the prediction performance of the linear spike-and-slab model with the GP based model and DepInfeR. We applied the proposed linear model to the same datasets used in Sec **??**, **??** and **??**, and compared its performance with DepInfeR and the GP-based model under the same simulation strategy. Simulation results are reported in Sec 4.

2.3 L_2 regularization on regression parameter β_s

DepInfeR puts L_1 regularization (group-LASSO) on the regression parameters β to prevent over-fitting. Here we demonstrate how our proposed model implicitly put L_2 regularization on the regression parameters. In particular, we show how the hyper parameter c_0 plays the role of the penalty parameter in a Ridge regression, and controls the shrinkage of β_s for $s = 1, ..., S$. To see this, note that by Equation (7), the full conditional posterior of β_s can be written as

$$p(\beta_s | Z, \alpha, \beta_{-s}, \sigma^2, X, Y_{obs}, c_0) \propto \mathcal{N}\left(Y_{-M_s}; \alpha_s \mathbf{1}_{D-|M_s|} + X_{-M_s}(Z * \beta_s), \sigma^2 I_{D-|M_s|}\right) \times \mathcal{N}\left(\beta_s; \mathbf{0}_P, c_0^{-1} \sigma^2 I_P\right) \tag{19}$$

for all $s = 1, ..., S$. Let $Y_s' = Y_{-M_s} - \alpha_s \mathbf{1}_{D-|M_s|}$, $X_s' = X_{-M_s} \text{diag}(Z)$. Equation (19) implies that the corresponding log posterior density takes the form

$$\log p(\beta_s | Z, \alpha, \beta_{-s}, \sigma^2, X, Y_{obs}, c_0) =_c -\frac{1}{2\sigma^2}\left(||(Y_s' - X_s'\beta_s)||_2^2 + c_0 ||\beta_s||_2^2\right), \tag{20}$$

where $=_c$ means equality up to a constant. From Equation (20) we see the Gaussian prior on β_s plays the role of the L_2 penalty in Ridge regression, and c_0 controls the strength of the shrinkage. As in [1], we recommend choosing the hyper parameter c_0 using cross validation.

2.4 Prediction performance of the linear model

In this section, we compare the prediction accuracy of the linear spike-and-slab model with the GP-based model and DepInfeR under the same simulation strategy described in the main manuscript. To examine the effect of imputation and data transformation, for each of the datasets, we apply the linear model to three data combinations: the original $\{X_{imp}, Y_{imp}\}$, $\{X_{imp}, Y_{logit}\}/\{X_{imp}, Y_{log}\}$, and the incomplete pair $\{X_{miss}, Y_{logit}\}/\{X_{miss}, Y_{log}\}$.

For each combination of dataset considered above, we also run a similar model without the protein selection step (i.e. fix the binary variables $z_p = 1$ for all $p = 1, \ldots, P$). Recall that unlike DepInfeR, our spike-and-slab linear model decouples the protein selection step and regularization step. Therefore we are interested in checking if including all proteins in the linear regression model would improve the prediction performance under an appropriate level of regularization. The results are reported in Fig 12, 13 and 14. Form the simulation results we see the linear model tends to outperform DepInfeR, but does not perform as well as the GP-based model.

3 A Synthetic example

Here we demonstrate the effectiveness of our proposed GP and linear spike-and-slab models using a synthetic example. We construct our synthetic dataset as follows: Let $D = 250, S = 40, P = 35$. We first set the synthetic drug-affinity matrix X to be a $D \times P$ matrix with all its entries being i.i.d. samples from $\text{Unif}(0, 1)$. To mimic the missing pattern in a real dataset, we first simulate four $D \times P$ matrices as described above, then randomly mask 0%(no), 30%(low), 50%(mid), and 70%(high) of the entries in them, and finally record the four resulting matrices as the synthetic drug-affinity matrices at different missing levels.

We then consider the following 4 basis functions defined on the domain $x \in (0, 1)$:

$$f_1(x) = 1.5x, \quad f_2(x) = -2x, \quad f_3(x) = \frac{1 - \exp(5x)}{1 + \exp(5x)}, \tag{21}$$

$$f_4(x) = 0.3 * \mathbf{1}(x > 0.3) + 0.7 * \mathbf{1}(x > 0.7). \tag{22}$$

We visualize the basis functions in Fig 7. For each of the synthetic drug-affinity matrices, each drug $d = 1, \ldots, D$ and each cell line $s = 1, \ldots, S$, we set Y_{ds}, the synthetic response of drug-cell line pair ds to be

$$Y_{ds} = \alpha_s + \sum_{p=1}^{10} \mathbb{1}(X_{dp} \text{ not missing})(\pi_{ps} f_{a_{ps}}(X_{dp}) + (1 - \pi_{ps}) f_{b_{ps}}(X_{dp})) + \epsilon_{ds}, \tag{23}$$

where X_{dp} is the corresponding entry in the synthetic drug-affinity matrix, $\alpha_s \sim \mathcal{N}(0, 1)$, $a_{ps}, b_{ps} \sim Unif(\{1, 2, 3, 4\})$, $\pi_{ps} \sim Unif(0, 1)$ for $p = 1, ..., 10$, and $\epsilon_{ds} \sim \mathcal{N}(0, 0.25)$. Let $Y = \{Y_{ds}\}_{d,s=1}^{D,S}$ be the resulting $D \times S$ synthetic drug-sensitivity matrix. Note that in this setup, only the first 10 columns of the synthetic drug-affinity matrix X are relevant to the synthetic responses, and the remaining 25 columns in

X are non-informative. This is to mimic the sparse signals in the real datasets. In addition, for each of the synthetic drug-affinity matrices at different missing levels, we randomly mask the same portion of entries (0%, 30%, 50%, 70%) in the corresponding synthetic drug-sensitivity matrix Y. The procedure above results in four synthetic datasets $\{X, Y\}$ at four different missing levels.

To examine if the proposed methods are able to recover the truth, we apply both linear and GP-based methods to these synthetic datasets, and examine how well do the proposed methods recover the individual f_{ps} and identify the informative columns in X at different missing levels. In addition to the two proposed model, we also include a third GP-based spike-and-slab model *with the standard Gaussian RBF kernel* to demonstrate how the modified kernel improves the fitting of the model. We denote the GP-based model with the modified and the standard Gaussian RBF kernel GP_0 and GP respectively. The hyperparameters of all models are chosen based on grid search and 3-fold cross validation. Simulation shows that both linear and GP-based spike-and-slab models are able to accurately identify all 10 informative columns at all four missing levels. We also report the f_{ps} estimated by the linear and the GP-based models using the full synthetic dataset (i.e. no missing values) at $p = 1$ and $s = 1, \ldots, 9$ in Fig 8. We see the f_{ps} estimated by the GP_0 model are closer to the true ones than the f_{ps} estimated by the unconstrained GP and the linear model. Even though the unconstrained GP model does not always recover f_{ps} accurately, we see both GP_0 and GP are able to capture the trends and shapes of all f_{ps} correctly. This is not always the case for the linear model. The "shift" between the true f_{ps} and those estimated by the unconstrained GP model is likely due to identifiability problem stems from the additive structure and the unconstrained GP prior. Similar figures at missing level $30\%, 50\%, 70\%$ can be found in Fig 9, 10,11.

References

[1] Batzilla A, Lu J, Kivioja J, et al (2022) Inferring tumor-specific cancer dependencies through integrating ex vivo drug response assays and drug-protein profiling. PLoS Computational Biology 18(8):e1010438

[2] Gelman A, Carlin JB, Stern HS, et al (2013) Bayesian data analysis. CRC press

[3] Ishwaran H, Rao JS (2005) Spike and slab variable selection: Frequentist and bayesian strategies. The Annals of statistics 33(2):730–773

[4] Liu JS (1994) The collapsed gibbs sampler in bayesian computations with applications to a gene regulation problem. Journal of the American Statistical Association 89(427):958–966

[5] Mitchell TJ, Beauchamp JJ (1988) Bayesian variable selection in linear regression. Journal of the american statistical association 83(404):1023–1032

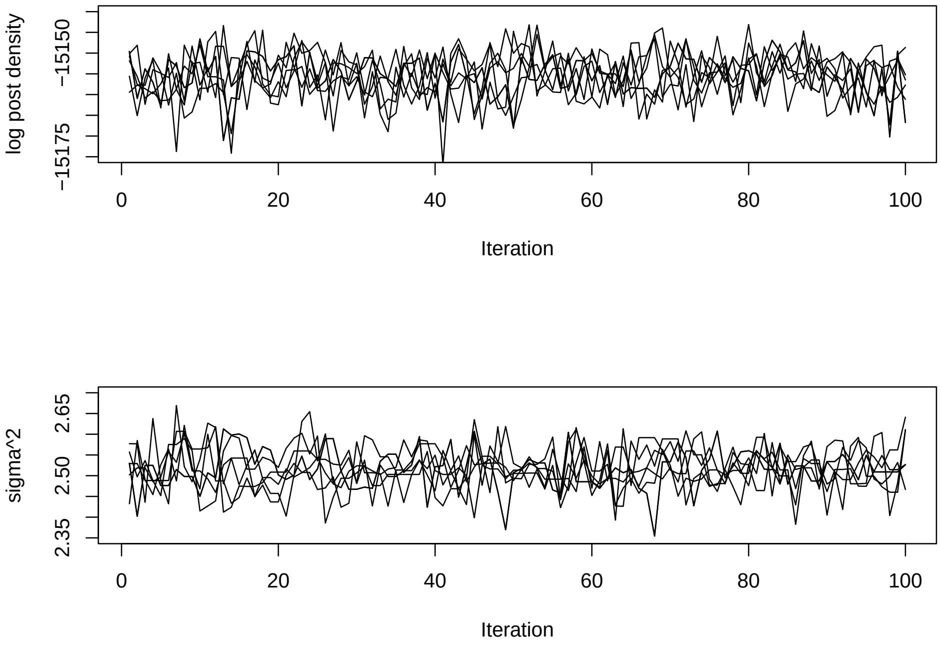

Fig. 2 Trace plots of the unnormalized log posterior density and σ^2 of the proposed model based on 6 repeated runs with GDSC1 dataset $\{X_{miss}, Y_{logit}\}$ and random initialization.

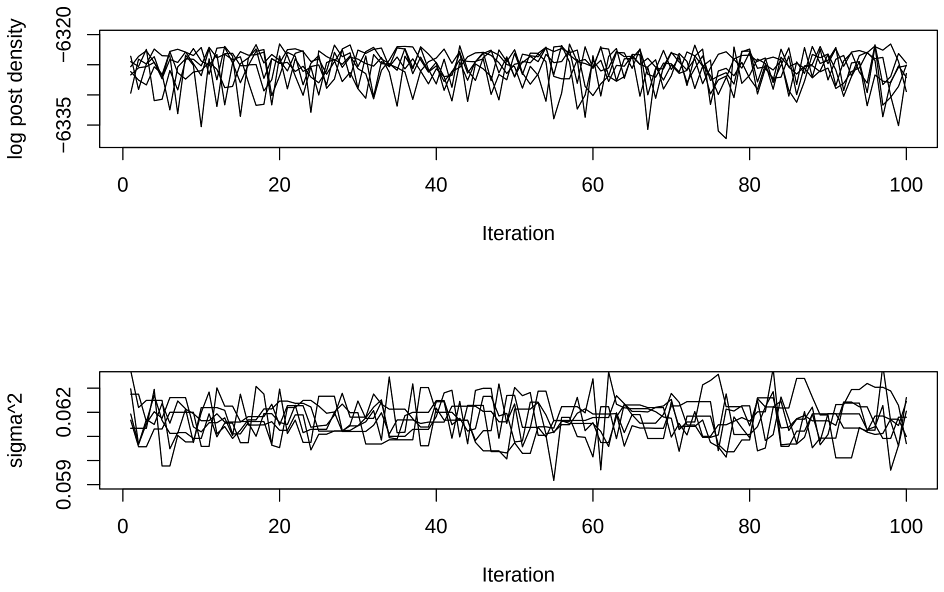

Fig. 3 Trace plots of the unnormalized log posterior density and σ^2 of the proposed model based on 6 repeated runs with beatAML dataset $\{X_{miss}, Y_{log}\}$ and random initialization.

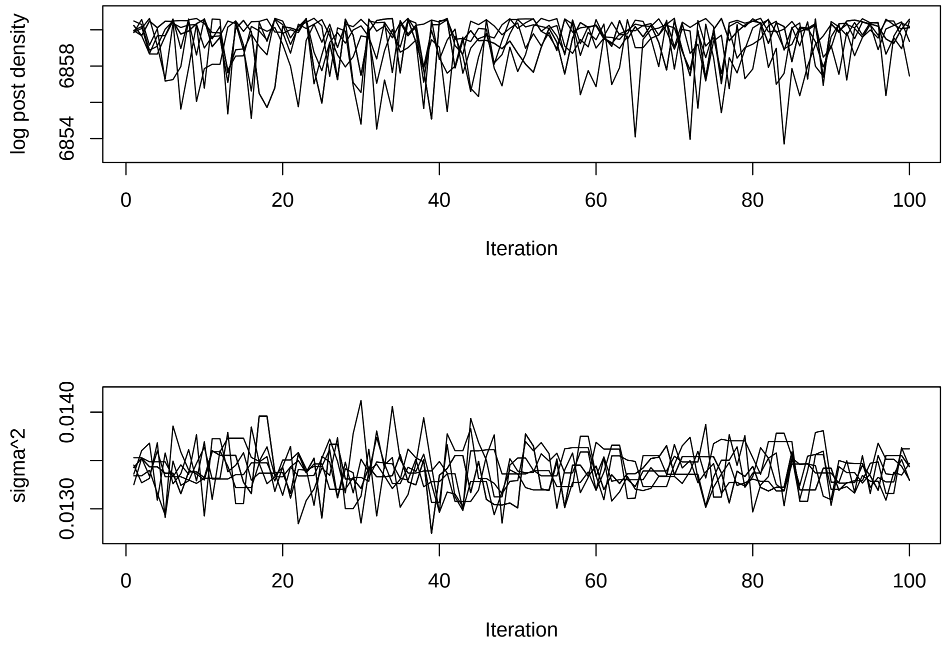

Fig. 4 Trace plots of the unnormalized log posterior density and σ^2 of the proposed model based on 6 repeated runs with EMBL dataset $\{X_{miss}, Y_{log}\}$ and random initialization.

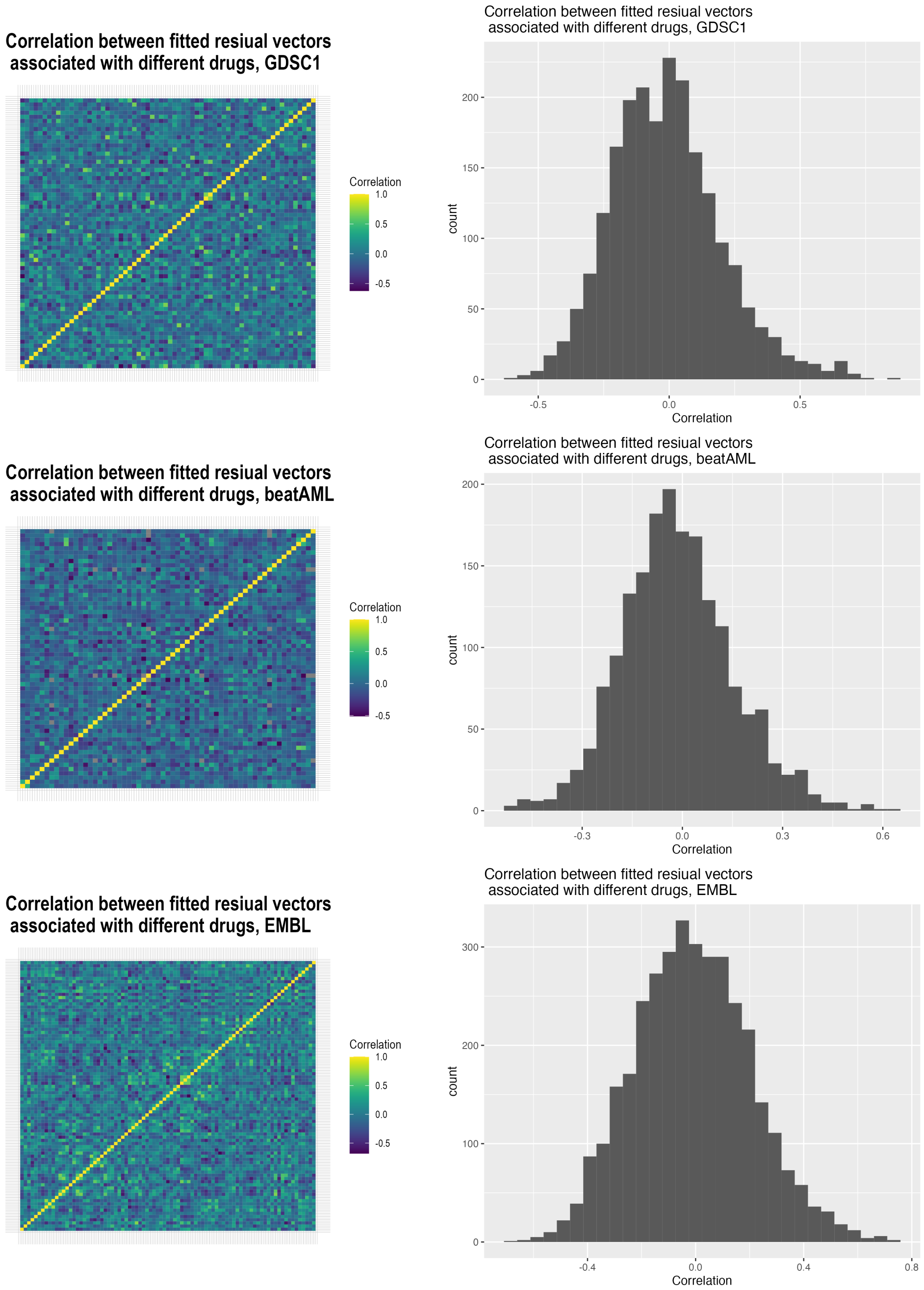

Fig. 5 Left: Heatmaps of the correlation matrices, each entry in the correlation matrix corresponds to the correlation between the fitted residual vectors of two drugs. Right: Histogram of the below-diagonal entries of the correlation matrix on the left.

A

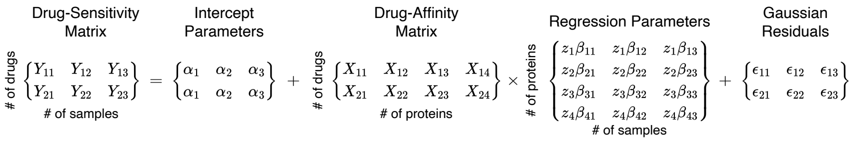

B

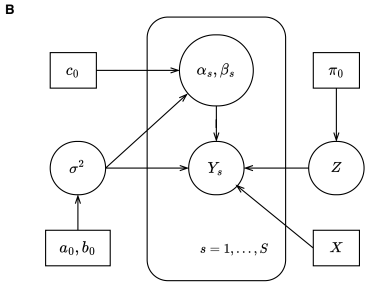

C

Table of variables		
Name	Distribution	Description
a_0, b_0	Constant	Hyperparameters of σ^2
c_0	Constant	Hyperparameter of α_s, β_s
π_0	Constant	Hyperparameter of Z
X	Constant	Processed drug-protein affinity matrix
σ^2	Inverse-Gamma	Scale parameter controlling the variance of the noises
α_s	Normal	Intercept parameter of the sth sample
β_s	Multivariate Normal	Regression parameters of the sth sample
Z	Bernoulli	Indicator variables controlling the inclusion of proteins
Y_s	Multivariate Normal	The sth column of the processed drug sensitivity matrix

Fig. 6 **A**: The proposed Spike-and-Slab linear regression model in matrix form. Here we assume $D = 2,\ S = 3,\ P = 4$. **B**: Graphical representation of the proposed Spike-and-Slab linear regression model. **C**: A table of all variables used to define the proposed model.

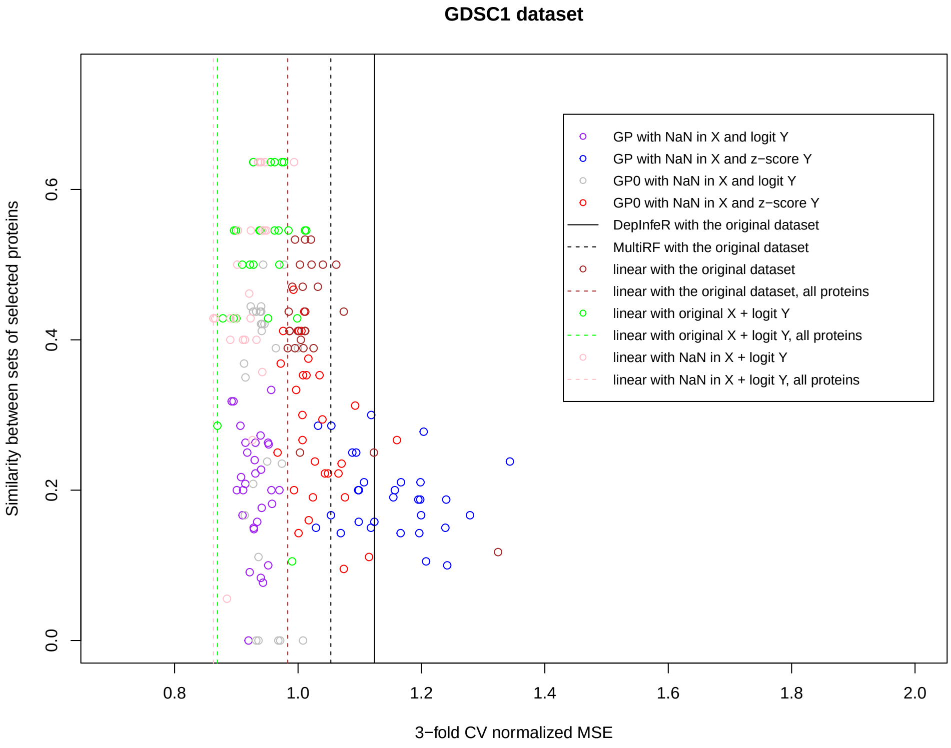

Fig. 7 **GDSC1** results. Each point corresponds to a model fitted under a given value of hyper-parameter. The horizontal coordinate of the point is the normalized MSE of the model estimated using 3-fold CV, and the vertical coordinate is the Intersection-over-Union score between the set of protein selected by the fitted model and the set of protein selected by DepInfeR. The vertical dashed and solid black lines correspond to the estimated normalized MSE of multivariate Random Forest and DepInfeR based on the original dataset $\{X_{imp}, Y_{imp}\}$.

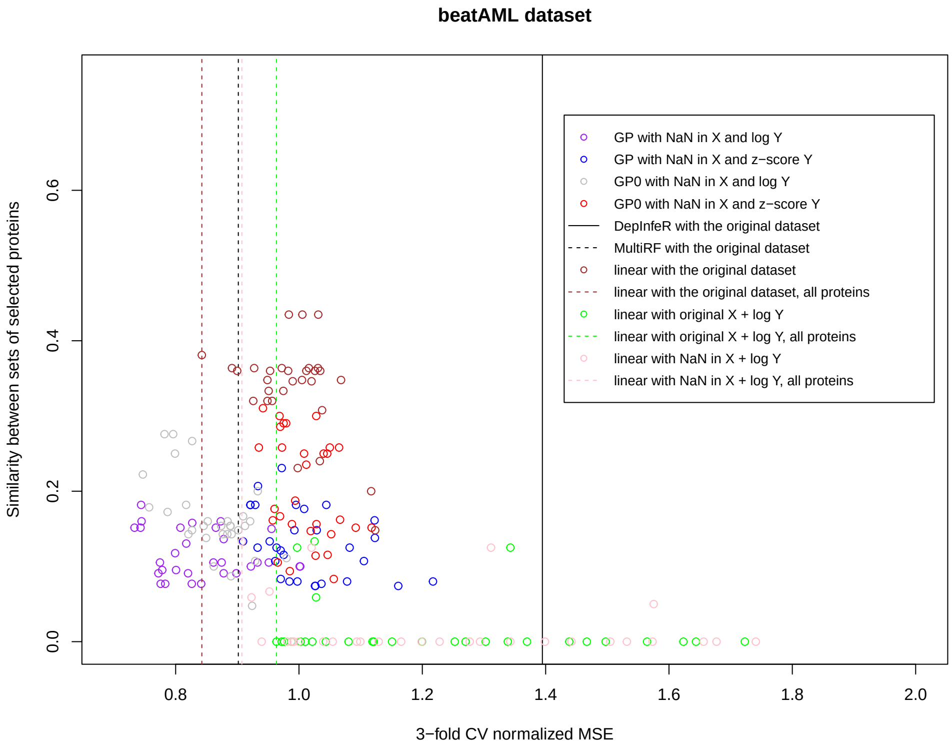

Fig. 8 beatAML results. Lines and points have the same interpretation as in Fig 12.

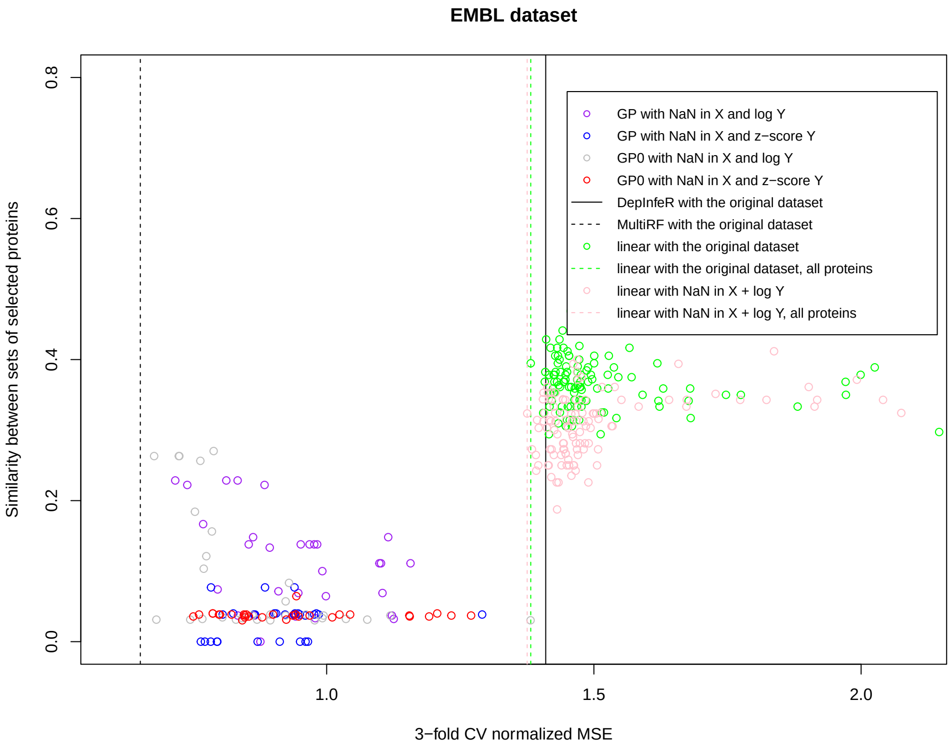

Fig. 9 **EMBL results**. Lines and points have the same interpretation as in Fig 12

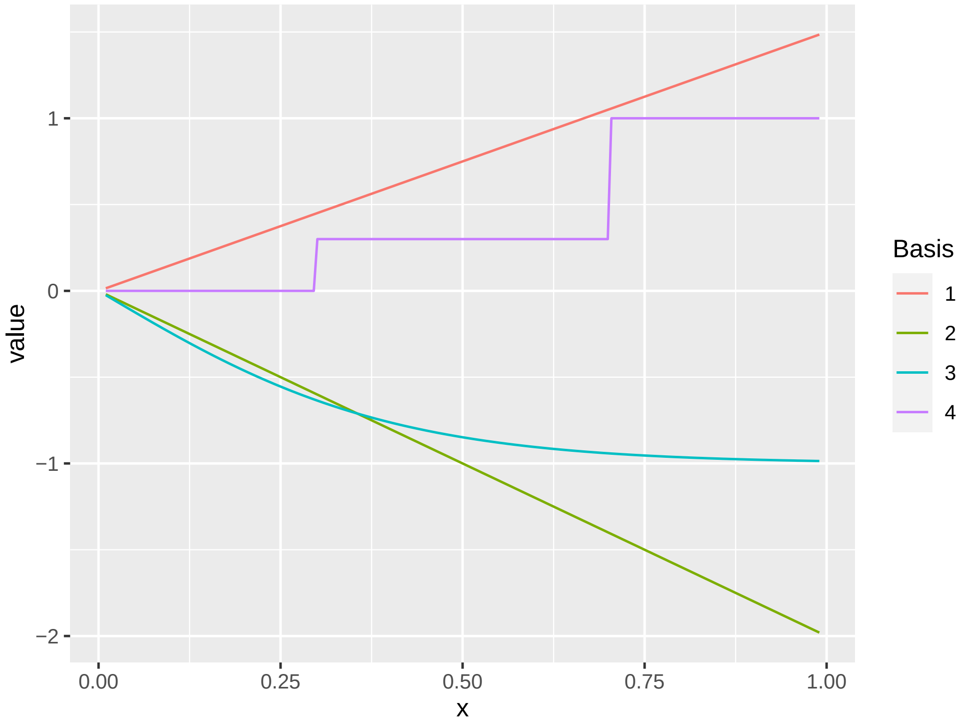

Fig. 10 **Synthetic Data Experiment.** Plots of the basis functions $\{f_i\}_{i=1}^4$

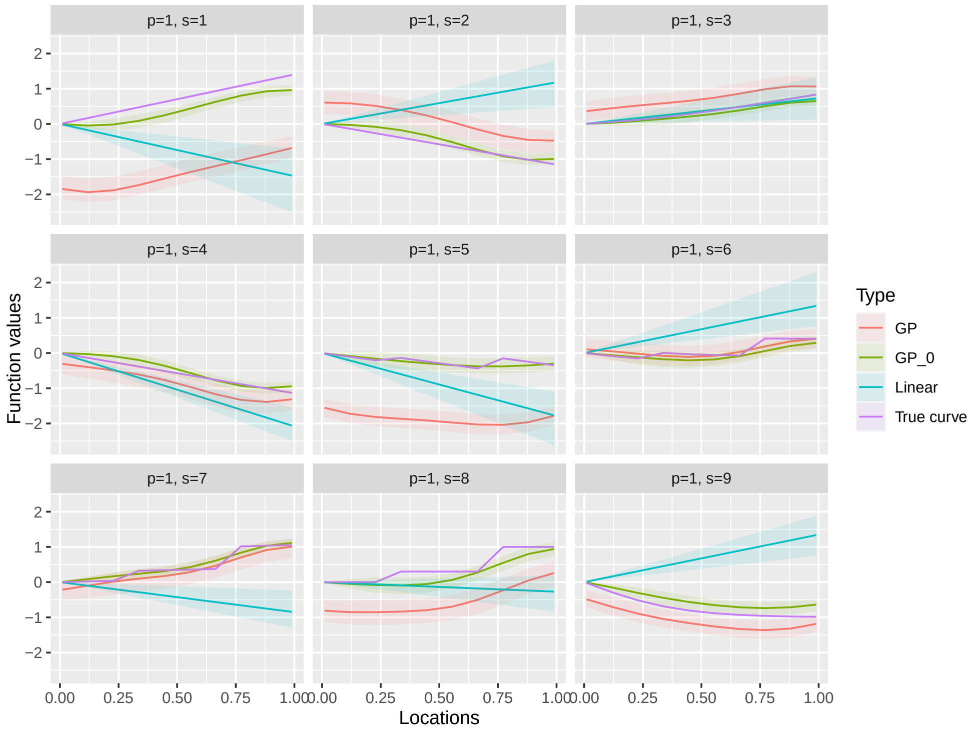

Fig. 11 f_{sp} for $p = 1$, $s = 1, \ldots, 9$ estimated based on the full dataset without any missing values. The shaded regions correspond to the 95% credible band of the estimated mean functions.

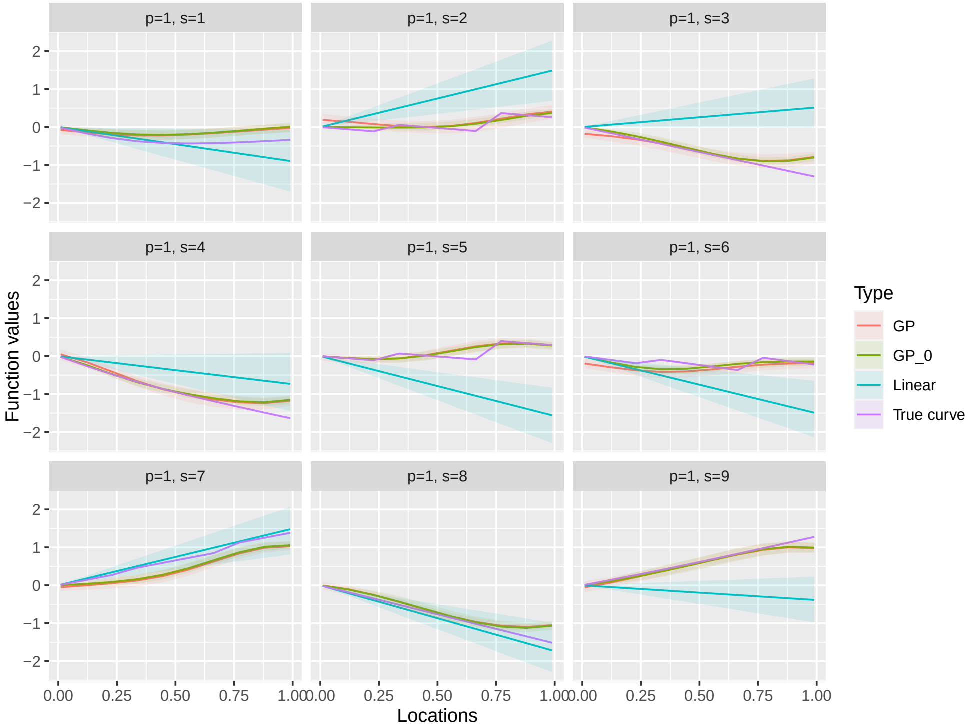

Fig. 12 f_{sp} for $p = 1$, $s = 1, \ldots, 9$ estimated based on the dataset with missing level 30%. The shaded regions correspond to the 95% credible band of the estimated mean functions.

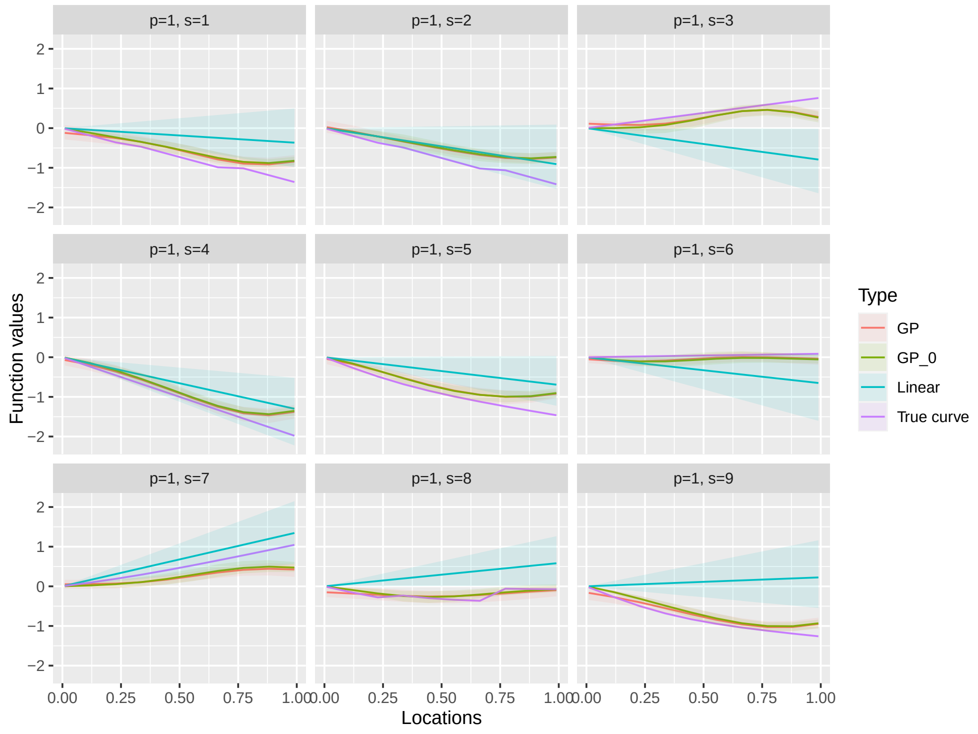

Fig. 13 f_{sp} for $p = 1$, $s = 1, \ldots, 9$ estimated based on the dataset with missing level 30%. The shaded regions correspond to the 95% credible band of the estimated mean functions.

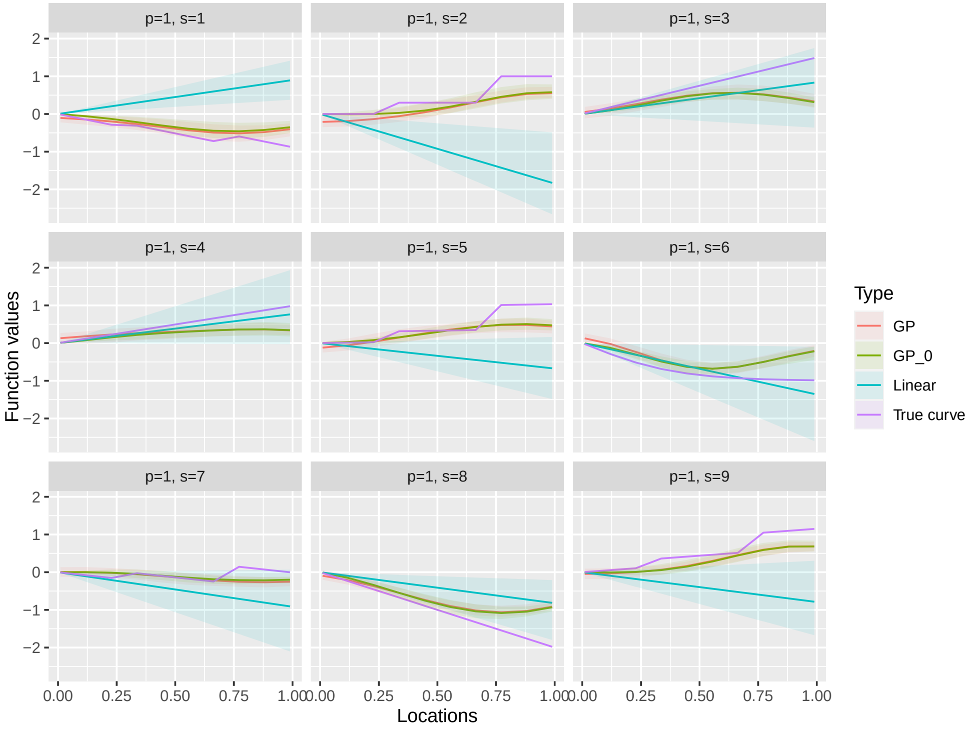

Fig. 14 f_{sp} for $p = 1$, $s = 1, \dots, 9$ estimated based on the dataset with missing level 30%. The shaded regions correspond to the 95% credible band of the estimated mean functions.
